# Cellular and molecular characterisation of the peripheral immune environment in migraine

**DOI:** 10.1097/PR9.0000000000001317

**Published:** 2025-08-20

**Authors:** Jayden A. O'Brien, Benjamin Heng, Seong Beom Ahn, Ananda Staats-Pires, Ashleigh Wake, Rebecca Wong, Aimie L. Peek, Noemi Meylakh, Karl Ng, Richard Stark, Vaughan G. Macefield, Helen M. McGuire, Luke A. Henderson, Paul J. Austin

**Affiliations:** a Brain and Mind Centre, School of Medical Sciences, Faculty of Medicine and Health, The University of Sydney, Sydney, NSW, Australia; b Macquarie Medical School, Faculty of Medicine and Health Sciences, Macquarie University, Sydney, NSW, Australia; c Baker Department of Cardiometabolic Health, University of Melbourne, Melbourne, VIC, Australia; d Department of Neurology and Neurophysiology, Royal North Shore Hospital, St Leonards, NSW, Australia; e Department of Neuroscience, Monash University, Melbourne, VIC, Australia; f Department of Neurology, The Alfred Hospital, Melbourne, VIC, Australia; g Charles Perkins Centre, School of Medical Sciences, Faculty of Medicine and Health, The University of Sydney, Sydney, Australia

**Keywords:** Chronic pain, CyTOF, Headache, Inflammation, Monocyte, Neuroimmune, Neutrophil, Platelet-leukocyte aggregate, Th17, Type 3 immunity

## Abstract

**Introduction:**

Peripheral immune dysfunction may be critically involved in the pathophysiology of migraine. Some evidence supports a role for peripheral T cells, monocytes, and humoral factors including kynurenine metabolites and cytokines, however a comprehensive picture has yet to emerge.

**Objective:**

This study sought to undertake a systematic assessment of the immune changes in episodic and chronic migraine across phases of the migraine cycle.

**Methods:**

Migraine patients in different phases of the migraine cycle with a confirmed diagnosis of episodic or chronic migraine and age- and sex-matched healthy controls were recruited. Peripheral blood was assessed for circulating immune cells, plasma proteins, and kynurenine pathway metabolites in a cross-sectional case–control design. Data were acquired using high-dimensional approaches including proteomics, single-cell mass cytometry, and imaging flow cytometry.

**Results:**

Plasma proteins related to increased cell–cell adhesion and altered enzymatic activity were increased in migraine. The migraine prodrome displayed a strong and distinct proinflammatory phenotype defined by increased platelet-neutrophil aggregation, quinolinic acid production, and matrix metalloproteinase-9 expression. Migraine patients in the attack phase instead expressed higher levels of cytokine receptors and phosphorylated transcription factors in Th17 cells, monocytes, natural killer cells, and B cells. T cells were shifted to a mobilised, recirculating phenotype across all migraine phases. Episodic and chronic migraine patients were only distinguished by subtle changes in T-cell phenotype.

**Conclusion:**

Distinct proinflammatory peripheral signatures were detected between migraine phases, while few alterations distinguished episodic and chronic status. These data provide a resource that may aid in the identification of peripheral immune cells and mediators contributing to migraine attack onset.

## Abstract 2

Supplemental Digital Content is Available in the Text.

An inflammatory peripheral blood immune environment, including platelet-neutrophil aggregation, matrix metalloproteinase 9 expression, Th17 activation, and quinolinic acid production, distinguishes migraine prodrome and attack.

## 1. Introduction

Migraine is the leading cause of disability in women under the age of 50.^3^ Despite advancements like anti-calcitonin gene–related peptide immunotherapy, migraine still imposes significant personal and economic burdens, and the complexities of the underlying pathophysiology remain to be fully elucidated. In particular, the mechanisms underlying transitions between the 4 phases of the migraine cycle—attack (ictus), prodrome (preictal), postdrome (postictal), and the pain-free period (interictal)—have yet to be identified.

The peripheral immune system likely plays a role in migraine pathology through interactions with the cerebral vasculature, trigeminal neurons, and meninges.^7^ Changes in circulating immune cells are observed in individuals with migraines, including CD4^+^ and CD8^+^ T cells, regulatory T cells (Tregs), and neutrophils, but the directionality of these changes is inconsistent and controversial.^2,10,11,22,24,28,31^ Differences in cell adhesion molecules on circulating lymphocytes,^25,35^ increased platelet-leukocyte aggregates (PLAs), particularly with neutrophils,^48^ and PLA-mediated leukocyte activation^33^ are also reported. Inflammatory and vascular proteins are also differentially modulated between painful and pain-free periods.^42^ One such example is matrix metalloproteinase 9 (MMP-9), which is upregulated by nitric oxide and proinflammatory cytokines and increases in blood during and after migraine attacks.^17,23^ Critically, MMP-9 degrades extracellular matrix and transiently upregulates intracellular inflammatory signalling and immune cell extravasation.^6,8^ Ictal increases in kynurenine pathway metabolites, such as anthranilic acid and 5-hydroxy-indoleaceticacid have been reported, while the neurotoxic quinolinic acid (QUIN) is increased in chronic pain conditions more broadly.^12,43^

A comprehensive analysis of cellular and molecular changes in the blood of migraine patients across different phases has yet to be performed. Such an analysis could provide crucial insights into the proinflammatory changes that initiate and resolve migraine attacks.

To address this, we performed a detailed profiling study of the peripheral blood immune environment in individuals with migraine using mass and imaging flow cytometry, proteomics, kynurenine pathway metabolite analysis, and enzyme-linked immunosorbent assays (ELISA). We hypothesised that migraine would be associated with increases in CD8^+^ T cells, classical monocytes, PLAs, changes in T-cell effector function, and upregulation of the p38 MAPK-MK2-NFκB axis as we have observed in other chronic pain conditions.^19,29,34^ We also hypothesised phase-dependent differences in short-lived neutrophils and platelets, stable T-cell signatures associated with activation and memory across the migraine cycle, and increased proinflammatory proteins, QUIN, and MMP-9.

## 2. Materials and methods

### 2.1. Ethics approval and consent to participate

All experiments were approved by the University of Sydney Human Research Ethics Committee (HREC #2021/083). Participants provided written informed consent. Data were handled in accordance with institutional policy and government legislation to protect privacy and confidentiality.

### 2.2. Participant information

Migraine patients (n = 49, female = 42) and healthy controls (n = 40, female = 32) were recruited between 2021 and 2023. Inclusion criteria were participants over 18 years of age with or without current analgesic use. Exclusion criteria included pregnancy and nonmigraine neurological conditions, including neuropathic pain and fibromyalgia, but not chronic back or neck pain. Migraine status was confirmed by a neurologist using the International Classification of Headache Disorders 3 (ICHD-3)^13^ and additional questionnaires on headache characteristics, pain intensity, visual disturbances, and psychosocial measures. Individuals with migraine were classified as chronic (n = 29) or episodic (n = 20) using ICHD-3 criteria (chronic: for more than 3 months, the incidence of more than 15 headache days per month of which at least 8 days have migraine features), and further divided into migraine phases (interictal: n = 13, episodic/chronic = 10/3; preictal: n = 14, e/c = 5/9; ictal: n = 10, e/c = 2/8; postictal: n = 8, e/c = 2/6; undetermined: n = 4) based on a combination of symptoms recorded in diary data, questionnaires, and interviews. Participants were designated as ictal if they reported headache with migraine features at time of blood draw; preictal if participants reported prodromal symptoms and had a confirmed migraine attack up to 72 hours later; postictal if postdromal symptoms were reported and a migraine attack occurred within 72 hours prior; and interictal if no symptoms of migraine prodrome, attack, or postdrome were present. Due to increased frequency of attacks, there were more preictal- and ictal-phase chronic than episodic migraine participants ($\chi_{3}^{2}$ = 9.66, *P* = 0.022). Reported clinical data scores were collected on the day of blood draw.

### 2.3. Blood collection

A single blood sample was drawn from each participant by a trained phlebotomist into ethylenediaminetetraacetic acid collection tubes and gently inverted. An aliquot of blood was fixed with proteomic stabilizer (PROT1; Smart Tube, Inc., NC0618275, distributed by ThermoFisher Scientific, Scoresby, VIC) and stored at −80°C for later cytometric analysis. Plasma was extracted from remaining blood and stored at −80°C. Due to ethical considerations, there was no drug wash-out; detailed summary drug information is reported in Supplementary Fig. 1, http://links.lww.com/PR9/A333.

### 2.4. Proteomics

Plasma samples from healthy controls (n = 10) and migraine participants (n = 10; 6 episodic, 4 chronic; 3 interictal, 2 preictal, 1 ictal, 2 postictal, 2 undetermined phase) underwent proteomic analysis by SWATH-mass spectrometry (SWATH-MS) at the Australian Proteome Analysis Facility, Macquarie University, Australia. Acquisition parameters are presented in Supplementary Methods 1, http://links.lww.com/PR9/A333. Proteins were considered differentially expressed if *P* < 0.05 and fold change > ±1.5 per previous methods.^30,47^ Gene set enrichment analysis was performed using gene ontology (GO) molecular function and biological process databases in EnrichR.^21^

### 2.5. Kynurenine metabolite analysis

Serum levels of xanthurenic acid (XA), tryptophan (TRP), kynurenine (KYN), 3-hydroxykynurenine (3-HK), 3-hydroxyanthranilic acid (3-HAA), anthranilic acid (AA), neopterin (NEO), kynurenic acid (KYNA), QUIN, and picolinic acid (PIC) were determined by ultra-high-performance liquid chromatography (uHPLC) or gas chromatography-mass spectrometry (GC/MS). Detailed acquisition parameters for each metabolite are presented in Supplementary Methods 2, http://links.lww.com/PR9/A333.

### 2.6. ELISA

Plasma levels of MMP-9 were analysed by ELISA (ABclonal; RK00217, distributed by Genesearch, Yatala, QLD) in chronic (n = 29) and episodic (n = 20) migraine patients and healthy controls (n = 31). Target abundance was quantified colorimetrically at 450 nm using a CLARIOstar plate reader (BMG Labtech, Mount Eliza, Victoria, Australia), and the final quantification for each sample was taken as the mean of 2 technical replicates.

### 2.7. Immunophenotyping by mass cytometry

Single-cell immunophenotyping of circulating immune cells from migraine patients (n = 49) and healthy controls (n = 40) was performed using mass cytometry. The full staining protocol is available in Supplementary Methods 3, http://links.lww.com/PR9/A333 and the reagents and antibodies in Supplementary Tables 1-2, http://links.lww.com/PR9/A333. Antibody optimisations demonstrated expected staining patterns on positive controls (Supplementary Figs. 2–4, http://links.lww.com/PR9/A333). Samples were acquired by cytometry by time-of-flight (CyTOF) on a Helios suspension mass cytometer.

### 2.8. Manual gating

CyTOF data were manually analysed by gating in FlowJo (v10.9, BD). The gating strategy is outlined in Supplementary Fig. 5, http://links.lww.com/PR9/A333. Signal intensities were normalised to EQ beads using the *premessa* R package (https://github.com/ParkerICI/premessa). All sample tubes were pregated for singlets and debarcoded in FlowJo and then blinded using the blind_files Python package (https://github.com/pokey/blind_files). The gating strategy is adapted from previous studies and identifies major leukocyte subsets, circulating endothelial cells, and cell activation states.^29,34^ Two controls (n = 2) were excluded from analyses involving RORγt expression due to high erythrocyte contamination.

### 2.9. Unsupervised analysis

The TidyTOF package^18^ was used to separately analyse pregated CD3^+^, CD19^+^, and CD3^−^CD19^−^ populations. Raw integrated ion counts were arcsinh-transformed (f = 5) and batch-corrected by both site and staining/acquisition round by linear rescaling. Cells were then clustered using the TidyTOF implementation of FlowSOM^44^ (parameters in Supplementary Table 3, http://links.lww.com/PR9/A333). Cluster identification was determined using heatmaps and ridgeline plots. Duplicate clusters were merged, and clusters containing doublets or heterogeneous populations were removed. Differential abundance analysis was conducted using one-way repeated measures ANOVA, with FlowSOM metacluster as the within-subjects term, followed by Holm-corrected pairwise *t*-tests. Differential expression analysis for activation markers was conducted using the TidyTOF implementation of diffcyt's limma method^45^ to α = 0.05.

### 2.10. Imaging flow cytometry of platelet-leukocyte aggregates

Imaging flow cytometry was used to quantify and visualise platelet-neutrophil aggregation.^15,16^ Migraine (n = 6 per phase) and healthy control (n = 12) fixed whole blood samples were similarly processed as for mass cytometry staining and immunostained for CD66b, CD61, CD62P, and MMP-9 (Supplementary Methods 4, http://links.lww.com/PR9/A333). Reagent details are provided in Supplementary Table 4, http://links.lww.com/PR9/A333. Data were acquired on an Amnis ImageStream X (3–5 × 10^5^ nonbead events per sample).

Data were gated using IDEAS software (Amnis) and compensated using single-colour–stained cells. Spatial parameters used to delineate coincident platelets and platelet-neutrophil aggregates were identified using the feature finder wizard and manually verified before inclusion in the gating strategy (Supplementary Fig. 6C, http://links.lww.com/PR9/A333). Data were excluded from the final analysis if they failed initial quality control for image or staining quality (n = 2) or if the clear isolation of specific gates or features failed (n = 2).

### 2.11. Data analysis

All *n* denote true biological replicates (participants), with single-cell measures from each participant aggregated by mean for cell type abundance or median for cellular marker expression. For any comparisons comparing migraine phases, migraine participants with an undetermined migraine phase (n = 4) were excluded. Statistical analysis was performed using *R* (ver. 4.4.0) in RStudio. Type 1 error rate was controlled by adjustment for multiplicity of comparisons. Continuous data were normality tested using per-group Shapiro–Wilk tests. Normally distributed data were assessed with 2-tailed unpaired *t*-tests or with 1-way ANOVA with *post hoc* pairwise *t*-tests and Holm multiple comparisons correction. Nonnormally distributed data were evaluated by Kruskal–Wallis test with *post hoc* pairwise two-signed Wilcoxon tests with Holm multiple comparisons using the *rstatix* package. *Post hoc* results are only reported where the main or interaction effect was statistically significant. Significance was evaluated to α = 0.05.

### 2.12. Data availability

Deidentified CyTOF data files (.fcs) are publicly available at Flow Repository (http://flowrepository.org/id/FR-FCM-Z85R). Additional deidentified analyses and raw data are available on the Open Science Framework (https://osf.io/npv9b/). The supplementary materials, http://links.lww.com/PR9/A333 contain further information and data useful for replicating the results.

## 3. Results

### 3.1. Demographic and questionnaire data

The proportion of female to male participants was not different between migraine phases (5 × 2 Fisher exact test, *P* = 0.32) nor between control, episodic, and chronic migraine groups (3 × 2 Fisher exact test, *P* = 0.82), nor was the mean age across groups or between sexes (2-way ANOVA; group × sex: *F*_*2,83*_ = 0.50; group: *F*_*2,83*_ = 0.61; sex: *F*_*1,83*_ = 0.35; all *P*_*adj*_ > 0.99). Clinical information and questionnaire scores are summarised in Table 1 and Supplementary Fig. 1, http://links.lww.com/PR9/A333.

**Table 1:** Summary of participant clinical information between control and migraine cohorts.

	Control	Migraine
n	40	49
n (female)	32	42
Age	32.9 ± 12.0	35.7 ± 12.64
Pain at time of blood draw (n)	1	22****
Drugs taken in the last 24 h (n)	7	29****
MIDAS	2.7 ± 4.3	69.9 ± 74.3****
HIT-6	41.6 ± 5.4	64.0 ± 7.5****
DASS depression	2.3 ± 3.0	8.6 ± 9.24***
DASS anxiety	0.89 ± 1.6	7.5 ± 7.6***
DASS stress	4.1 ± 4.6	12.5 ± 8.6***

Values are mean ± standard deviation or n values as appropriate.

***P < 0.001; ****P < 0.0001.

DASS, depression, anxiety, and stress score; HIT-6, headache impact test 6; MIDAS, migraine disability score.

### 3.2. Proteomic analysis of plasma detected cell adhesion and enzymatic alterations

**Figure 1.**
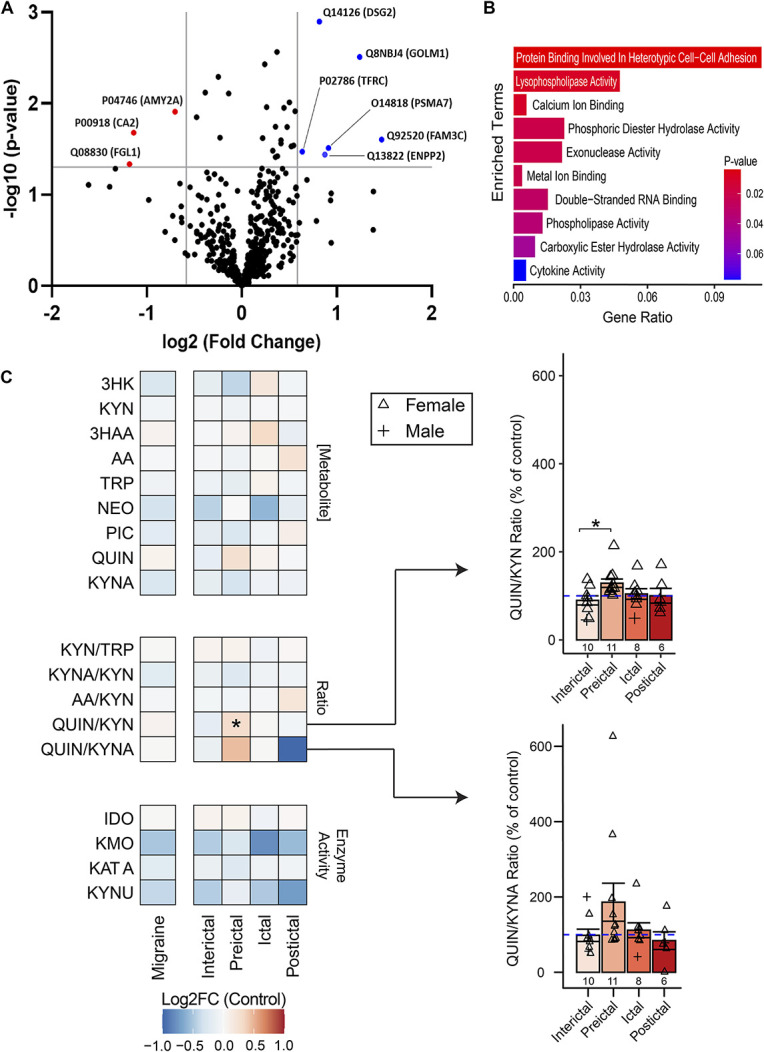
Differential proteomic analysis of human plasma between a preliminary sample of migraine patients and healthy controls. (A) Proteins identified via proteomic analysis with a greater than 1.5-fold change and a *P*-value <0.05. Positive log2FC values indicate upregulation in individuals with migraine, and negative values indicate downregulation. FAM3C: proteome FAM3C/interleukin-like EMT inducer; GOLM1: Golgi membrane protein 1/GP73; PSMA7: proteasome subunit alpha type-7/RC6-1/XAPC7; ENPP2: ectonucleotide pyrophosphatase/phosphodiesterase family member 2/autotaxin; DSG2: desmoglein-2; TFR1: transferrin receptor protein 1/CD71; AMYP: pancreatic alpha-amylase; CAH2: carbonic anhydrase 2; FGL1: fibrinogen-like protein 1. (B) Gene ontology (GO) enrichment analysis on differentially expressed genes (>1.5-fold change and *P* < 0.05) using GO biological process terms revealed a significant overrepresentation of proteins related to cell adhesion, lipid metabolism, and intracellular signalling. Enrichment of cytokine activity–related proteins did not reach significance. Healthy control: n = 10, migraine: n = 10 (episodic n = 6, chronic n = 4). (C) Log_2_-fold change of kynurenine metabolite concentrations, ratios, and calculated enzymatic activities compared to controls. The QUIN/KYN ratio was specifically significantly increased in the preictal phase compared to the interictal phase. The QUIN/KYNA ratio followed a similar trend, but there were no significant differences. Column graphs represent group mean ± standard error. *P < 0.05. 3HAA, 3-hydroxyanthranilic acid; 3HK, 3-hydroxykynurenine; AA, anthranilic acid; IDO, indoleamine 2,3-dioxygenase; KAT A, kynurenine aminotransferase A; KMO, kynurenine 3-monooxygenase; KYN, kynurenine; KYNA, kynurenic acid; KYNU, kynureninase; NEO, neopterin; PIC, picolinic acid; QUIN, quinolinic acid; TRP, tryptophan.

For an initial assessment of circulating inflammatory changes in migraine, mass spectrometry-based proteomics was performed on a preliminary cohort. Six proteins were significantly upregulated in migraine with a >1.5 fold change compared to controls and *P*_*adj*_ < 0.05 (FAM3C, GOLM1, PSA7, ENPP2, DSG2, TFRC); 3 proteins were significantly downregulated (FGL1, CAH2, AMYP; Fig. 1A). Gene set enrichment analysis revealed a statistically significant overrepresentation of proteins involved in heterotypic cell–cell adhesion (*P* = 0.004), lysophospholipase activity (*P* = 0.009), calcium ion binding (*P* = 0.010), and phosphodiesterase activity (*P* = 0.02; Fig. 1B). This suggests the differential modulation of cell–cell interactions, lipid metabolism, and intracellular signalling in migraine.

### 3.3. Peripheral inflammatory signatures vary strongly between migraine phases

#### 3.3.1. Kynurenine pathway metabolites demonstrate a preictal neurotoxic signature

The dysregulation of the kynurenine pathway and its neurotoxic metabolite (QUIN) have been implicated in the pathogenesis of neuropathic pain^27,32,39^ and migraine.^20^ Plasma levels of all major kynurenine pathway metabolites were quantified (Fig. 1C). The QUIN to KYN ratio, a measure of the proportion of metabolism along the neurotoxic branch of the pathway, was significantly increased in the preictal phase compared to the interictal phase (*H*_*3*_ = 7.87, *P* = 0.049; *post hoc P*_*adj*_ = 0.045). The QUIN to KYNA ratio, a measure of the balance of neurotoxic to neuroprotective metabolites, was not significantly different between migraine phases (*H*_*3*_ = 5.79, *P* = 0.12), although followed a similar trend to the QUIN/KYN ratio. No other comparisons showed significant differences. These results together reflect a neurotoxic kynurenine metabolic environment specific to the preictal phase of migraine.

#### 3.3.2. Platelet-neutrophil aggregation distinguishes the migraine preictal period

**Figure 2.**
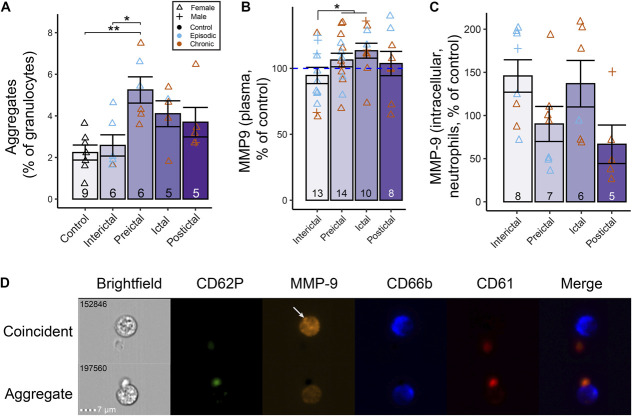
Platelet-neutrophil aggregation increases in the preictal period alongside perturbations in intracellular neutrophil MMP-9 expression. (A) The frequency of platelet-neutrophil aggregates, as a proportion of all granulocytes, was significantly elevated in preictal the phase compared to the interictal phase and healthy controls. (B) Plasma MMP-9 levels were increased in the preictal and ictal phases compared to the interictal phase. Data are normalised to healthy controls. (C) Neutrophil intracellular MMP-9 expression as measured by mass cytometry was not significantly different between migraine phases. Data are normalised to healthy controls. (D) Imaging flow cytometry analysis was used to investigate platelet-neutrophil aggregation and to verify MMP-9 expression results. Shown are a representative “coincident” event (top row), in which a CD66b^+^MMP-9^hi^ neutrophil and a CD61^+^CD62P^+^ platelet are present in the same frame but are not directly adjacent to each other, and an “aggregate” event (bottom row) in which the neutrophil and platelet are confirmed to be physically interacting. The white arrow indicates an example MMP9^+^ granule within a neutrophil. Column graphs represent group mean ± standard error. CD62P, P-selectin; MMP-9, matrix metalloproteinase-9. *P < 0.05; **P < 0.01.

Platelet-neutrophil aggregation, as measured by imaging flow cytometry, was significantly increased preictally (*F*_*4,26*_ = 5.30, *P* = 0.003) compared to the interictal period (*P*_*adj*_ = 0.015) and healthy controls (*P*_*adj*_ = 0.003), with an approximately two-fold increase in the proportion of neutrophils with platelets adhered to its surface (Fig. 2A). This proportion then gradually decreased over the migraine cycle. The frequencies of singlet neutrophils and platelets were not significantly different (neutrophils: *F*_*4,26*_ *=* 1.61*, P* = 0.20; platelets: *F*_*4,26*_ *=* 0.66*, P* = 0.63), suggesting that aggregation is not driven by increased production of these cells (Supplementary Fig. 6A-B, http://links.lww.com/PR9/A333). These results demonstrate a clear increase in platelet-neutrophil aggregation in the hours preceding migraine attack.

Total plasma MMP-9 levels were increased in the preictal and ictal phases compared to the interictal phase (*F*_*1,35*_ = 4.57, *P* = 0.04; Fig. 2B). The effect of MMP-9 expression across migraine phases did not reach statistical significance (*F*_*3,22*_ = 2.81, *P* = 0.063), although there was a clear trend of a decrease in the preictal and postictal phases compared to the interictal and ictal phases (Fig. 2C). Imaging flow cytometry provided visual verification of true platelet-neutrophil aggregation and intracellular localisation of MMP-9 to the cytoplasm with pronounced expression in granular structures (Fig. 2D; Supplementary Fig. 6C-E, http://links.lww.com/PR9/A333).

#### 3.3.3. T cells express phase-stable recirculation markers and phase-specific transcription factor phosphorylation

**Figure 3.**
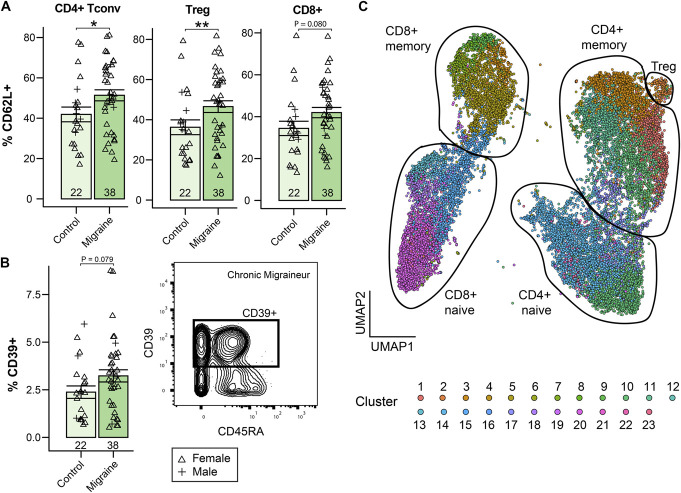
CD4^+^ T cells upregulate markers related to lymph node recirculation in migraine. (A) The percentage of CD4^+^ conventional (Tconv) and regulatory (Treg) T cells positive for the recirculation marker CD62L was significantly increased in migraine. The difference was not significant in CD8^+^ T cells. (B) CD39^+^ T cells were slightly, but not significantly, increased in migraine. Biaxial plot shows a representative gate from a chronic migraine patient. (C) UMAP dimensionality reduction of the 23 FlowSOM-derived clusters used for unsupervised analysis. *P < 0.05; **P < 0.01. UMAP, uniform manifold approximation and projection.

CD3^+^ cells were analysed by both manual and unsupervised analysis. Manual analysis showed no difference in the CD4:CD8 ratio (*H*_*4*_ = 3.16, *P* = 0.53; Supplementary Fig. 7D, http://links.lww.com/PR9/A333). There were T-cell–wide increases in the expression of CD62L, a recirculation-promoting T-cell surface protein, that was significant in CD4^+^ conventional (Tconv; *t*_*43.5*_ = −2.08, *P* = 0.044) and regulatory T cells (Treg; *z* = 248, *P* = 0.009), but not CD8^+^ T cells (*t*_*41.9*_ = −1.80, *P* = 0.080), in individuals with migraine compared to healthy controls (Fig. 3A). Frequencies of naïve, effector/Temra, central memory, and effector memory subsets were not significant in any T-cell group (all *P* > 0.05). An increase in the proportion of CD39^+^ T cells in migraine compared to controls did not reach significance (*z* = 303, *P* = 0.079; Fig. 3B).

**Figure 4.**
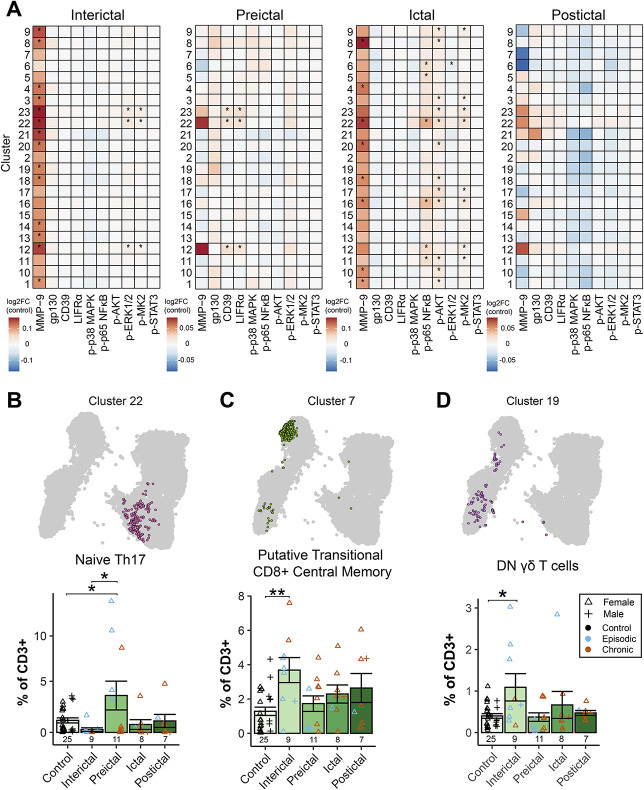
Differential abundance and expression analysis of T-cell clusters reveals phase-specific and phase-stable changes related to the migraine disease state. (A) Differential expression analysis of activation markers found a near-global upregulation of MMP-9 expression in circulating T cells in the interictal phase and demonstrate a specific upregulation of phospho-ERK1/2 and phospho-MK2 in cluster 12 (memory Th17), cluster 22 (naïve Th17), and cluster 23 (Tc17), which are each associated with type 3 immunity. In the preictal phase, CD39 and LIFRα were upregulated specifically in Th17 and Tc17 subsets. The ictal phase, compared to healthy controls, showed upregulated MMP-9 in many T-cell clusters alongside phosphorylated p65 NFκB, AKT, and MK2. Phospho-ERK1/2 was decreased in a CD8^+^ central memory T-cell cluster. There were no differences detected in the postictal phase. (B) Unsupervised analysis revealed that the frequency of cluster 22 (naïve Th17 cells) was increased in the preictal phase compared to the interictal phase and healthy controls. (C) Cluster 7, a putative transitional CD8^+^ central memory T-cell subset, was more frequent in the interictal phase compared to controls. (D) The frequency of cluster 19 (double-negative γδ T cells) was increased in the interictal phase compared to controls. Column graphs represent group mean ± standard error. *P < 0.05; **P < 0.01. AKT, protein kinase B; ERK, extracellular signal–related kinase; gp130, glycoprotein 130; LIFRα, leukemia inhibitory factor receptor alpha; MAPK, mitogen-activated protein kinase; MK2, MAPK-activated protein kinase 2; MMP-9, matrix metalloproteinase-9; NFκB, nuclear factor kappa-B; STAT3, signal transducer and activation of transcription 3.

Unsupervised analysis identified 23 unique clusters across CD4^+^, CD8^+^, naïve, memory, and regulatory subsets (Fig. 3C, Supplementary Fig. 7A-B, Supplementary Table 5, http://links.lww.com/PR9/A333). The expression of activation markers, including MMP-9, IL-6 receptor family molecules, and phosphorylated transcription factors, significantly varied by migraine phase between clusters compared to healthy controls. In the interictal period, MMP-9 was upregulated in most clusters, and phospho-ERK1/2 and phospho-MK2 were significantly upregulated in type 3 immunity-associated T cells (memory CD4^+^ Th17, naïve CD4^+^ Th17, and CD8^+^ Tc17; clusters 12, 22, and 23 respectively). These clusters also expressed the highest levels of MMP-9. Individuals with migraine in the preictal period showed increased expression of the ectonucleotidase CD39 and the IL-6 receptor family subunit LIFRα in these type 3 T-cell subsets. The ictal period was associated with increased MMP-9, phospho-p65 NFκB, phospho-AKT, and phopsho-MK2 across many subsets. There was also a decrease in phospho-ERK1/2 in CD8^+^ central memory T cells (cluster 6). These results demonstrate distinct inflammatory signatures in different migraine phases, with the involvement of Th17 and Tc17 cells (Fig. 4A).

Differential abundance analysis of FlowSOM results revealed differences for 3 clusters (phase × cluster: *F*_*88,1188*_ = 1.649, *P* = 0.0002). The frequency of naïve Th17 cells (cluster 22) was elevated preictally compared to the interictal phase (*P*_*adj*_ = 0.021) and to healthy controls (*P*_*adj*_ = 0.044), further suggesting a role for type 3 immunity-associated T cells (Fig. 4B). “Transitional” CD8^+^ central memory T cells (cluster 7) were significantly more frequent in the interictal phase compared to healthy controls (*P*_*adj*_ = 0.003; Fig. 4C). Likewise, double-negative γδ T cells (cluster 19) were increased in the interictal phase compared to controls (*P*_*adj*_ = 0.023; Fig. 4D). Visualisations used to annotate CD3^+^ clusters are provided in Supplementary Fig. 7C, http://links.lww.com/PR9/A333, and full cluster annotations are available in Supplementary Table 5, http://links.lww.com/PR9/A333. Overall, these data indicate T cells undergo both phase-stable and phase-specific changes in migraine.

#### 3.3.4. Myeloid cells display an enhanced proinflammatory state in interictal and ictal phases

**Figure 5.**
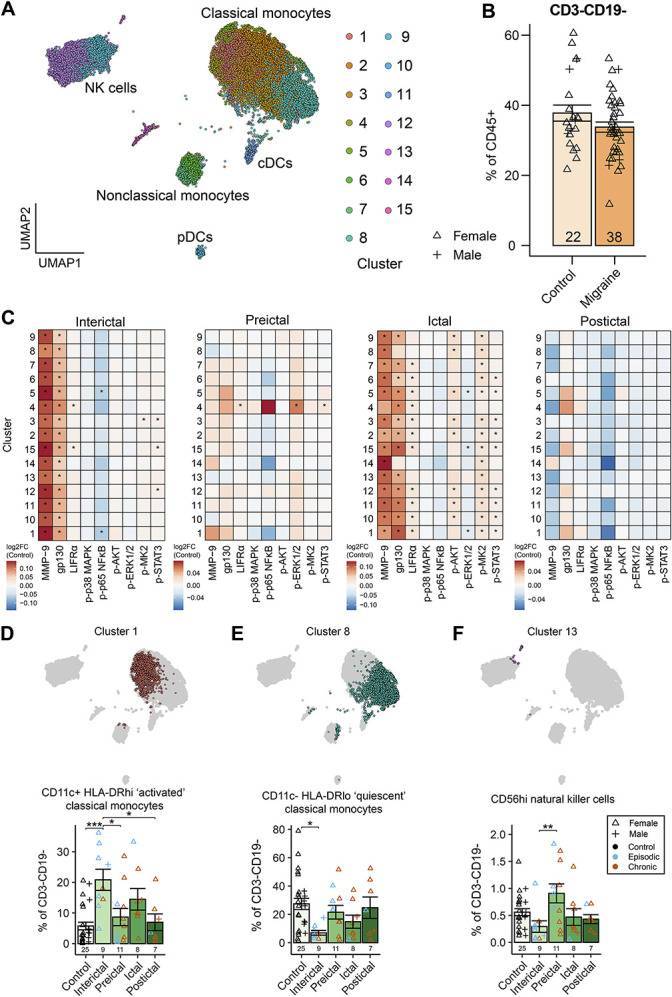
Myeloid lineage circulating cells polarise toward a proinflammatory state during migraine attack as measured by mass cytometry. (A) UMAP of CD3^−^CD19^−^ cells clustered using FlowSOM. (B) Manual gating analysis of CD3^−^CD19^−^ frequency as a proportion of all CD45^+^ cells found no significant difference between groups. (C) Differential expression analysis of FlowSOM clusters shows multiple differences between the migraine interictal period and healthy controls. MMP-9 and gp130 expression was significantly increased in all clusters. LIFRα was increased in clusters 4 and 15. Phospho-p65 NFκB expression was decreased in clusters 1 (HLA-DR^hi^CD11c^+^ “activated” classical monocytes) and 5 (CD14^+^CD34^+^ putative monocyte progenitors). Phospho-MK2 was increased in cluster 3 (CD133^hi^ classical monocytes) and phospho-STAT3 was increased in clusters 3, 12 (CD16^hi^ NK cells), and 15 (HLA-DR^-^CD62L^+^). In the preictal phase, cluster 4 (CD45RA^hi^ classical monocytes) showed increased expression of LIFRα, phospho-ERK1/2, and phospho-STAT3 compared to controls. In the ictal phase, MMP-9, gp130, and LIFRα were increased in most clusters, as were phosphorylated AKT, MK2, and STAT3. Phospho-ERK1/2 was decreased in clusters 1, 5, and 15. There were no significant differences in the postictal phase. (D) An increased proportion of “activated” classical monocytes (cluster 1) was observed in the interictal phase compared with preictal and postictal phases and healthy controls. (E) A proportional interictal decrease in “quiescent” classical monocytes (cluster 8) was observed compared to controls. (F) The frequency of CD56^hi^ NK cells (cluster 13) was significantly increased in the preictal phase compared to the interictal phase. Column graphs represent group mean ± standard error. AKT, protein kinase B; cDC, conventional dendritic cell; ERK, extracellular signal–related kinase; gp130, glycoprotein 130/CD130; LIFRα, leukemia inhibitory factor receptor subunit alpha; MAPK, mitogen-activated protein kinase; MK2, MAPK-activated protein kinase 2; MMP-9, matrix metalloproteinase-9; NFκB, nuclear factor kappa-B; NK, natural killer; pDC, plasmacytoid dendritic cell; STAT3, signal transducer and activation of transcription 3. *P < 0.05; **P < 0.01; ***P < 0.001. UMAP, uniform manifold approximation and projection.

Myeloid cell and natural killer (NK) cell data were analysed by manual and unsupervised analysis. Uniform manifold approximation and projection (UMAP) dimensionality reduction showed a clear separation of 5 major cell types (classical monocytes, nonclassical monocytes, conventional type 2 dendritic cells [cDC2], plasmacytoid dendritic cells, and NK cells) across 15 clusters (Fig. 5A). There were no significant differences in their proportions between migraine phases as a percentage of all CD45^+^ cells (Supplementary Fig. 8A-E, http://links.lww.com/PR9/A333) or in the myeloid compartment overall (Fig. 5B).

Unsupervised differential expression analysis of myeloid clusters across migraine phases showed significant upregulation of MMP-9 and the IL-6 receptor family subunit glycoprotein 130 (gp130 or CD130) across all clusters in the interictal period compared to controls, and LIFRα, phospho-MK2, and phospho-STAT3, and decreased phospho-p65 NFκB in select clusters. There were widespread changes in the ictal period, with upregulation of MMP-9, gp130, LIFRα, phospho-AKT, phospho-MK2, and phospho-STAT3 across many clusters and decreases in phospho-ERK1/2 in some clusters. In contrast, differences in preictal expression levels compared to controls were restricted to CD45RA^hi^ classical monocytes (Fig. 5C).

Differential abundance analysis of clusters showed strong effects of migraine phase (phase × cluster: *F*_*56,756*_ = 2.53, *P* < 0.0001), with 3 clusters having altered abundance across migraine phases. HLA-DR^hi^CD11c^+^ “activated” classical monocytes (cluster 1) were significantly increased in the interictal phase compared with preictal (*P*_*adj*_ = 0.013) and postictal phases (*P*_*adj*_ = 0.012) and with controls (*P*_*adj*_ = 0.00015), but not the ictal phase (*P*_*adj*_ = 0.57; Fig. 5D). A proportional interictal decrease in HLA-DR^lo^CD11c^−^ “quiescent” classical monocytes (cluster 8) was observed compared to controls (*P*_*adj*_ = 0.029; Fig. 5E). CD56^hi^ NK cells (cluster 13) were significantly increased in the preictal phase compared to the interictal phase (*P*_*adj*_ = 0.009; Fig. 5F). Visualisations used to annotate clusters are provided in Supplementary Fig. 8, http://links.lww.com/PR9/A333, and full cluster annotations are available in Supplementary Table 5, http://links.lww.com/PR9/A333. Together, these results support a baseline proinflammatory myeloid signature in the interictal phase of migraine, with intensification of cell activation states in the ictal phase.

CD19^+^ B cells expressed increased MMP-9, phospho-MK2 and decreased phospho-ERK1/2 during migraine attack across most clusters; there were no other significant differences in B-cell phenotype (Supplementary Fig. 9; Supplementary Table 5, http://links.lww.com/PR9/A333).

### 3.4. Episodic and chronic migraine differ subtly in unconventional and regulatory T-cell signatures

**Figure 6.**
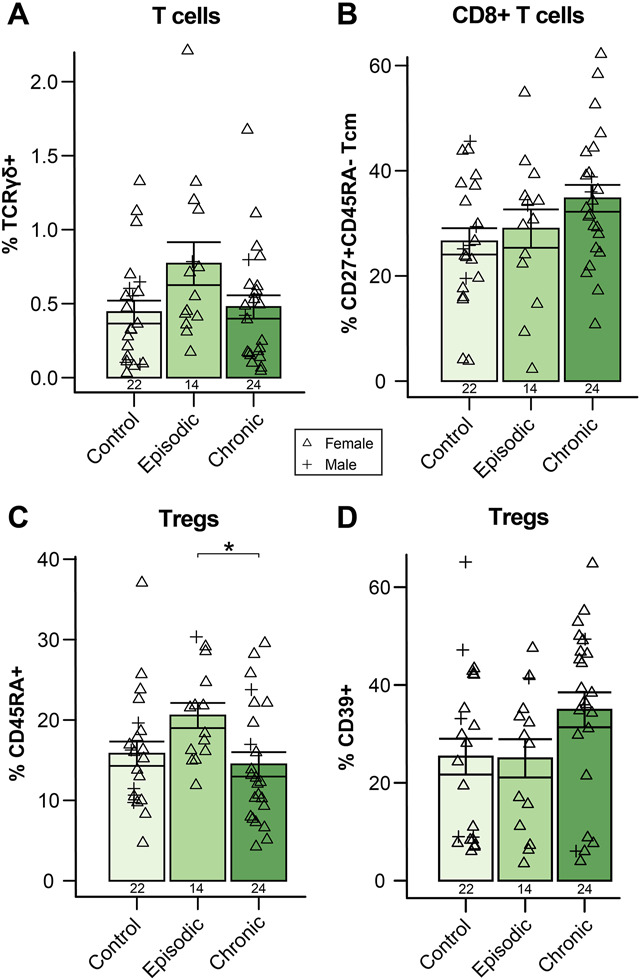
Subtle changes in T-cell phenotype may differentiate chronic and episodic migraine. (A) TCRγδ^+^ T cells, as a proportion of all CD3^+^ T cells, were slightly, but not significantly, increased in episodic migraine compared to chronic migraine and healthy controls. (B) The proportion of CD8^+^ T cells with a central memory phenotype was not significantly different between migraine frequencies but was slightly elevated in chronic migraine. (C) The proportion of naïve CD45RA^+^ Tregs was significantly lower in chronic compared to episodic migraine. (D) The proportion of regulatory T cells expressing CD39 was slightly, but not significantly, elevated in chronic compared to episodic migraine. Column graphs represent group mean ± standard error. Tcm, central memory T cell; Treg, regulatory T cell. *P < 0.05.

Alterations in immune cell proportions differentiating chronic and episodic migraine were not robustly observed except for largely nonsignificant changes in T-cell subsets. γδ T cells trended higher in episodic compared to chronic migraine and healthy controls (*H*_*2*_ = 4.77, *P* = 0.09; Fig. 6A). CD8^+^CD27^+^CD45RA^−^ central memory T cells trended higher in chronic migraine (*F*_*2,57*_ = 2.58, *P* = 0.085; Fig. 6B). CD45RA^+^ Tregs were significantly reduced in chronic compared to episodic migraine (*F*_*2,57*_ = 3.47, *P* = 0.038; chronic vs episodic *P*_*adj*_ = 0.037), suggesting an increased proportion of memory Tregs (Fig. 6C). The CD39^+^ Treg proportion trended higher in chronic migraine (*H*_*2*_ = 4.76, *P* = 0.09; Fig. 6D). No other differences between chronic and episodic migraine were detected.

## 4. Discussion

This study highlights distinct phase-dependent and phase-stable changes in peripheral immune cells, circulating proteins, and kynurenine metabolites that contribute to migraine pathophysiology. Between, migraine phases, we observed elevated platelet-neutrophil aggregation, a neurotoxic kynurenine metabolic environment, and proinflammatory transcription factor phosphorylation states in the migraine preictal period. Phase-specific increases were seen in the expression of MMP-9, IL-6 receptor subunits, and phosphorylated transcription factors in specific cell subsets. T-cell phenotypes exhibited phase-stable changes, with CD4^+^ conventional and regulatory T-cell subsets showing consistent polarisation toward CD62L^+^ and CD27^+^ states.

Importantly, the only differences detected between episodic and chronic migraine were subtle changes in unconventional and regulatory T-cell phenotype. The other measured parameters varied much more by phase independently of episodic or chronic status. This suggests that peripheral inflammatory factors are associated with the impending or current presence of migraine but are not directly responsible for the clinical features that distinguish episodic and chronic migraine. The regulatory T-cell differences detected between episodic and chronic migraine, however, warrant future study to clarify their meaning.

Proteomic analysis identified several differentially expressed proteins related to cell–cell adhesion and enzymatic activity. Notably, FAM3C, or interleukin-like epithelial-to-mesenchymal transition inducer (ILEI), is a cytokine that regulates extracellular matrix–receptor interactions, wound healing, and cell migration.^37,38^ ILEI also activates LIFR, triggering STAT3-related intracellular signalling pathways to drive epithelial-to-mesenchymal transition.^46^ The increased expression of LIFRα and STAT3 observed here in multiple cell types suggests a possible role for this pathway in migraine. ENPP2 (autotaxin) is a major generator of known pronociceptive metabolite lysophosphatidic acid (LPA), which is also known to strongly activate platelets.^5^ These proteins may be of interest for further investigation in the context of migraine to determine their true biological significance.

The kynurenine pathway, which involves enzymes that convert tryptophan into various metabolic products with inflammatory/neurotoxic or neuroprotective effects,^40^ was dysregulated. Quinolinic acid exerts multimodal neurotoxic and proinflammatory effects directly on neurons, glia, and on the permeability of nervous system microvasculature and was the most common biomarker of chronic pain in a study with 13,765 participants.^12^ Our findings support the possibility that kynurenine metabolism is deleteriously altered in the preictal phase with increased QUIN/KYN ratio contributing to migraine attack onset.

Neutrophil activation may be implicated in migraine pathophysiology due to their role in inhibition of T-cell proliferation, promotion of extracellular matrix remodelling, and contribution to vascular damage. In peripheral blood, neutrophils are the primary producers of MMP-9, which is a mediator of extracellular matrix degradation, angiogenesis, and chemokine-dependent leukocyte extravasation at the blood–brain barrier.^1^ Previous studies have noted increases in plasma MMP-9 during migraine attack, which we have replicated.^4,17,23^ Extending these findings, we have shown that MMP-9 expression by neutrophils may vary between migraine phases. This study also provides definitive evidence of increased platelet-neutrophil aggregation in the migraine preictal period. This is consistent with the involvement of platelets in migraine^9,41^ and is in line with observations of PLAs in previous flow cytometry studies.^49^

Increased CD62L on T cells and the expansion of a CD8^+^ central memory T-cell cluster interictally suggests polarisation toward central memory and naïve subsets across multiple T-cell compartments. CD62L expression is critical for lymphoid tissue homing, and its loss promotes cell migration to peripheral sites and effector activity. It should be investigated whether this may represent a compensatory mechanism that counterbalances peripheral T-cell meningeal infiltration and modulation of trigeminovascular function.

Type 3 immunity-associated T cells (Th17 and Tc17 cells) were expanded and activated in migraine. Th17 cells are implicated in several pain conditions, including low back pain, rheumatoid arthritis, neuropathic pain, and complex regional pain syndrome,^14,19,26^ suggesting a potential role in migraine that warrants further exploration. In addition, a shift in classical monocyte phenotypes toward a more activated, proinflammatory state was observed during the interictal and ictal periods, along with an expansion of CD56^hi^ NK cells preictally. Evidence of monocyte activation in migraine attack via NFκB has been previously reported^36^ and may represent an increased capacity to generate proinflammatory cytokines and to differentiate into macrophages in tissue.

### 4.1. Limitations

This study has some limitations. The cross-sectional, between-subjects study design may have introduced individual variation in phase-dependent measures that could not be controlled. Chronic migraine patients are inherently more likely to present in the preictal, ictal, and postictal phases than episodic patients, which makes it challenging to balance phase membership between these groups. Future studies with a within-subjects design would be ideal to replicate these findings. The low numbers of male participants precluded analysis of sex as a biological variable. The proteomic findings are based on a relatively small sample size, which prevented stratification of results by migraine frequency or phase. Finally, while many transcription factors were differentially phosphorylated in the mass cytometry data, log-fold changes were typically small. Their pathological relevance, therefore, requires further assessment.

## 5. Conclusions

This study supports the involvement of complex interactions between immune, vascular, and metabolic pathways in migraine. Some of these changes may better reflect the phase in the migraine cycle than episodic/chronic migraine status. Further investigation is required to validate these phase-dependent changes, link these changes to underlying mechanisms, and distinguish those involved in migraine pathogenesis from compensatory or resolution-related changes. This understanding may lead to the identification of biomarkers predictive of attack onset and improved therapeutic targets for people suffering from migraine.

## Disclosures

The authors declare that they have no competing interests.

## Supplemental digital content

Supplemental digital content associated with this article can be found online at http://links.lww.com/PR9/A333.
