# Supplementary material for: Cellular and molecular characterisation of the peripheral immune environment in migraine
Source: Pain Rep. 2025 Aug 20;10(5):e1317. doi: 10.1097/PR9.0000000000001317 (PMC12369775; doi:10.1097/PR9.0000000000001317)
Supplement: Supplementary file 1 [file painreports-10-e1317-s001.docx]

**Supplementary Materials**

| **Supplementary Methods 1-4:** | Pages 2-5 |
| --- | --- |
| **Supplementary Figures 1-9:** | Pages 6-16 |
| **Supplementary Tables 1-5:** | Pages 17-20 |

**Supplementary Methods 1. Proteomic SWATH-MS acquisition details.**

The data-dependent acquisition (DDA) method was employed to generate recombinant and human plasma spectral libraries, while the data-independent acquisition (DIA) method was used for SWATH-MS quantification analysis. Both methods were performed on a SCIEX TripleTOF 6600 (SCIEX, Framingham, MA) coupled with an Eksigent Ultra nanoLC system (Eksigent Technologies, Dublin, CA), following the procedures outlined in our previously published work.[1] Protein identification was performed on ProteinPilot Software with a human database from SwissProt (reviewed 20,422 human protein sequences, 2023 version), and SWATH data extraction was conducted using PeakView 2.1 using previously described parameters.[3]

**Supplementary Methods 2. Kynurenine metabolite uHLPC and GC/MS acquisition details.**

Levels of xanthurenic acid (XA), tryptophan (TRP), kynurenine (KYN), 3-hydroxykynurenine (3-HK), 3-hydroxyanthranilic acid (3-HAA) and anthranilic acid (AA) in deproteinised serum were determined by an uHPLC system (Agilent 1290 Infinity, CA, USA) with a sequential diode-array UV and fluorescence detection and Agilent ZORBAX Rapid Resolution High Definition C18, reversed phase column (2.1 x 150mm, 1.8μm, Agilent Technologies, CA, USA) as previously described.[2] The identification and quantification of XA, KYN and 3-HK were performed by a UV detector (G4212A, Agilent, CA, USA) with absorbance at 250nm and a reference signal at 350nm for XA; and with absorbance at 365nm and a reference signal off for KYN and 3-HK. The identification and quantification of TRP, 3-HAA and AA were performed by a fluorescence detector (G1321B xenon flash lamp, Agilent, CA, USA) with an emission wavelength of 438nm and an excitation wavelength of 280nm for TRP and 320nm for 3-HAA and AA.

Neopterin (NEO) concentrations in deproteinised serum samples were determined as previously described.[4] The identification of NEO was performed by a fluorescence detector (G1321B xenon flash lamp, Agilent, CA, USA) with an emission wavelength of 438nm and an excitation wavelength of 355nm. Kynurenic acid (KYNA) concentrations in serum samples were determined by HPLC (Agilent 1260 Infinity, Agilent, CA, USA) and an Agilent ZORBAX Rapid Resolution High Definition C18, reversed phase (4.6 x 100mm, 3.5μm, Agilent Technologies, CA, USA). Mobile phase consisted of 95% of 50mM sodium acetate and 50mM zinc acetate, pH 5.2 and 5% v/v HPLC grade acetonitrile. The flow rate was set at 1.00mL/min with an isocratic elution. The identification of KYNA was performed by a fluorescence detector (G1321B xenon flash lamp, Agilent, CA, USA) with emission wavelength of 388nm and an excitation wavelength of 344nm. Quinolinic acid (QUIN) and picolinic acid (PIC) concentrations in serum samples were determined using an Agilent 7890 gas chromatograph coupled with an Agilent 5975 mass spectrometer following a protocol previously described.[2] Results for all metabolites were calculated by interpolation using a 6-point calibration curve. Ratios between metabolites and/or enzyme activities were calculated as follows: indoleamine 2,3-dioxygenase (IDO): KYN/TRP×100; kynurenine 3-monooxygenase (KMO): 3HK/KYN; kynurenine aminotransferase A (KAT A): KYNA/KYN×100; kynureninase (KYNU): AA/KYN×100 + 3HAA/3HK×100.

**Supplementary Methods 3. CyTOF immunostaining.**

CyTOF immunostaining was completed in five batches, with one healthy control sample used as a between-batch reference control. Following each staining step, without washing, samples were topped up with 1mL of the relevant staining buffer and centrifuged at 600g for 5 min at 4˚C (before fix/perm) or 800g for 7 min at 4C (after fix/perm). Antibody incubations were done in 50µL per 2 million cells.

Stabilised blood samples underwent erythrocyte lysis according to the manufacturer’s instructions (Smart Tube, Inc). Pellets that were not the expected colour (pink-white) and size after four lysis steps were assessed under the microscope and discarded if cell recovery was poor and/or an unsuitably high level of debris was present.

Per participant, 2 × 10^6^ cells were passed on for staining, with pellets resuspended in 200µL of 100IU/mL porcine heparin in FACS buffer (0.5% w/v BSA, 2mM EDTA, 0.02% w/v sodium azide in sterile PBS) and incubated at RT for 20 min to reduce charge-based eosinophilic background staining.[5] Cells were then stained with 209Bi-CD45 in FACS buffer for 30 min on ice to measure *bona fide* CD45 expression, then barcoded with the relevant Pd-CD45 for 30 min on ice for participant identification. Cells were washed twice, then samples combined such that each new tube contained samples from one healthy control, episodic migraineur, and chronic migraineur, using 104Pd-CD45, 108Pd-CD45, and 110Pd-CD45. Cells were stained for surface antibodies for 30 min on ice, followed by 161Dy-anti-APC for 20 min on ice, then incubated in 1mL fixation/permeabilisation buffer at 4°C overnight.

The following day, cells were washed twice in FACS buffer and once in permeabilisation buffer, blocked with 200µL 100IU/mL heparin in permeabilisation buffer for 20 min at RT, and then incubated with intracellular antibodies in permeabilisation buffer for 30 min on ice and 164Dy-anti-biotin in permeabilisation buffer for 20 min on ice. Cells were washed once in permeabilisation buffer, once in FACS buffer, then once again in fix/permeabilisation buffer in preparation for nuclear staining with 100% ice-cold methanol, added dropwise, for 30 min on ice. Cells were then blocked in 200µL 100IU/mL heparin in FACS buffer for 20 min at RT and stained with nuclear antibodies in FACS buffer for 30 min on ice and 169Tm-anti-Cy5 for 20 min on ice. Each batch included a mass-minus-many (MMM) gating control in which intracellular and nuclear primary antibodies were excluded.[6] Finally, cells were fixed in 4% paraformaldehyde in PBS, pH 6.9, containing 1:4000 DNA-intercalator for 20 min at RT. For acquisition, cells were washed once each in FACS buffer, ultrapure water, and cell acquisition solution (CAS), then resuspended at 1 million cells/mL in 1:10 EQ beads in CAS and filtered into a 5mL round-bottom tube with cell strainer cap before acquisition.

**Supplementary Methods 4. Imaging flow cytometry immunostaining.**

Cells underwent erythrocyte lysis, and then were blocked in 1:5 human Fc receptor block (eBioscience, #14-9161-71) in fetal bovine serum-based flow cytometry stain buffer (BD Pharmingen, #554656) for 20 min on ice. Cells were then incubated with surface antibodies for 30 min on ice, incubated in fix/permeabilisation buffer (eBioscience) for 30 min at RT, washed twice in permeabilisation buffer, and then incubated with MMP-9-biotin primary antibody in permeabilisation buffer for 30 min on ice. The biotin was reacted with 1:200 streptavidin-Alexa Fluor 568 in permeabilisation buffer for 20 min on ice, then cells were washed twice in stain buffer and fixed in 200µL 4% PFA in PBS at 4˚C overnight. The following day, cells were washed thrice in stain buffer, then resuspended in residual volume prior to acquisition.

**Supplementary Method References**

[1] Ahn SB, Kamath KS, Mohamedali A, Noor Z, Wu JX, Pascovici D, Adhikari S, Cheruku HR, Guillemin GJ, McKay MJ, Nice EC, Baker MS. Use of a Recombinant Biomarker Protein DDA Library Increases DIA Coverage of Low Abundance Plasma Proteins. J Proteome Res 2021;20:2374–2389.

[2] Guillemin GJ, Cullen KM, Lim CK, Smythe GA, Garner B, Kapoor V, Takikawa O, Brew BJ. Characterization of the Kynurenine Pathway in Human Neurons. J Neurosci 2007;27:12884–12892.

[3] Kavyani B, Ahn SB, Missailidis D, Annesley SJ, Fisher PR, Schloeffel R, Guillemin GJ, Lovejoy DB, Heng B. Dysregulation of the Kynurenine Pathway, Cytokine Expression Pattern, and Proteomics Profile Link to Symptomology in Myalgic Encephalomyelitis/Chronic Fatigue Syndrome (ME/CFS). Mol Neurobiol 2024;61:3771–3787.

[4] de Paula Martins R, Ghisoni K, Lim CK, Aguiar AS, Guillemin GJ, Latini A. Neopterin preconditioning prevents inflammasome activation in mammalian astrocytes. Free Radic Biol Med 2018;115:371–382.

[5] Rahman AH, Tordesillas L, Berin MC. Heparin reduces nonspecific eosinophil staining artifacts in mass cytometry experiments. Cytometry A 2016;89:601–607.

[6] Takahashi C, Au-Yeung A, Fuh F, Ramirez-Montagut T, Bolen C, Mathews W, O’Gorman WE. Mass cytometry panel optimization through the designed distribution of signal interference. Cytom Part J Int Soc Anal Cytol 2017;91:39–47.


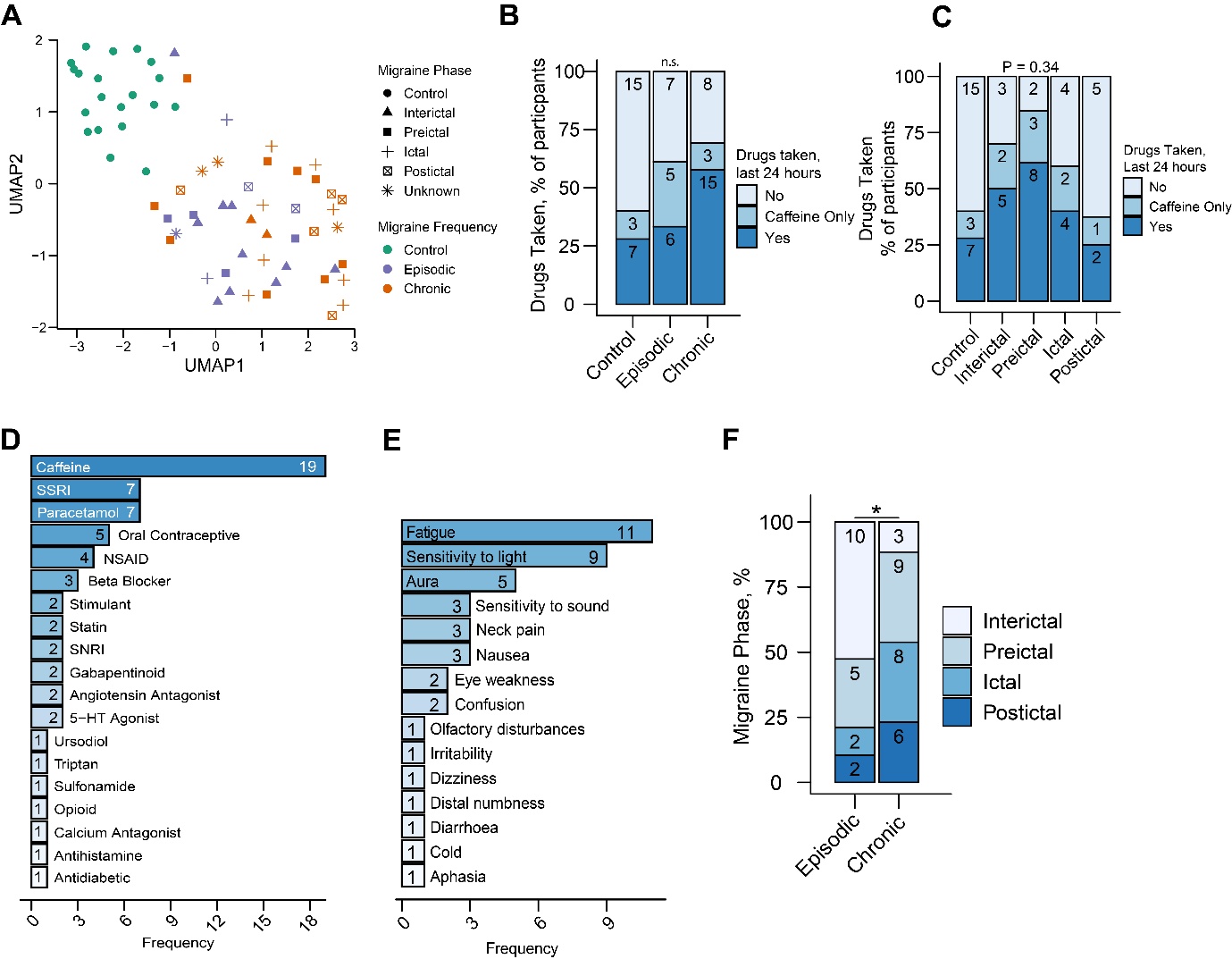


**Supplementary Figure 1**. Additional participant clinical information. **(A)** UMAP dimensionality reduction of clinical scores demonstrates separation between control and migraineur participants. There were no clear groupings by migraine frequency or phase. Each dot represents one participant. **(B)** The proportion of participants taking prescription or other drugs within 24 hours before blood draw was not significantly different between migraine frequencies (χ^2^_4_ = 7.85, *P* = 0.097), though it was highest in chronic migraineurs. (**C)** The proportion of participants who had taken medications within the 24 hours prior to blood draw was not significantly different (*χ^2^_8_* = 9.00, *P* = 0.342), but highest for those in the preictal period. **(D)** Frequency of specific drug classes used by participants in the study across both controls and migraineurs. **(E)** Frequency of specific prodromal symptoms reported by migraineurs. **(F)** There were more preictal- and ictal-phase chronic than episodic migraineurs (*χ^2^_3_* = 9.66, *P* = 0.022).


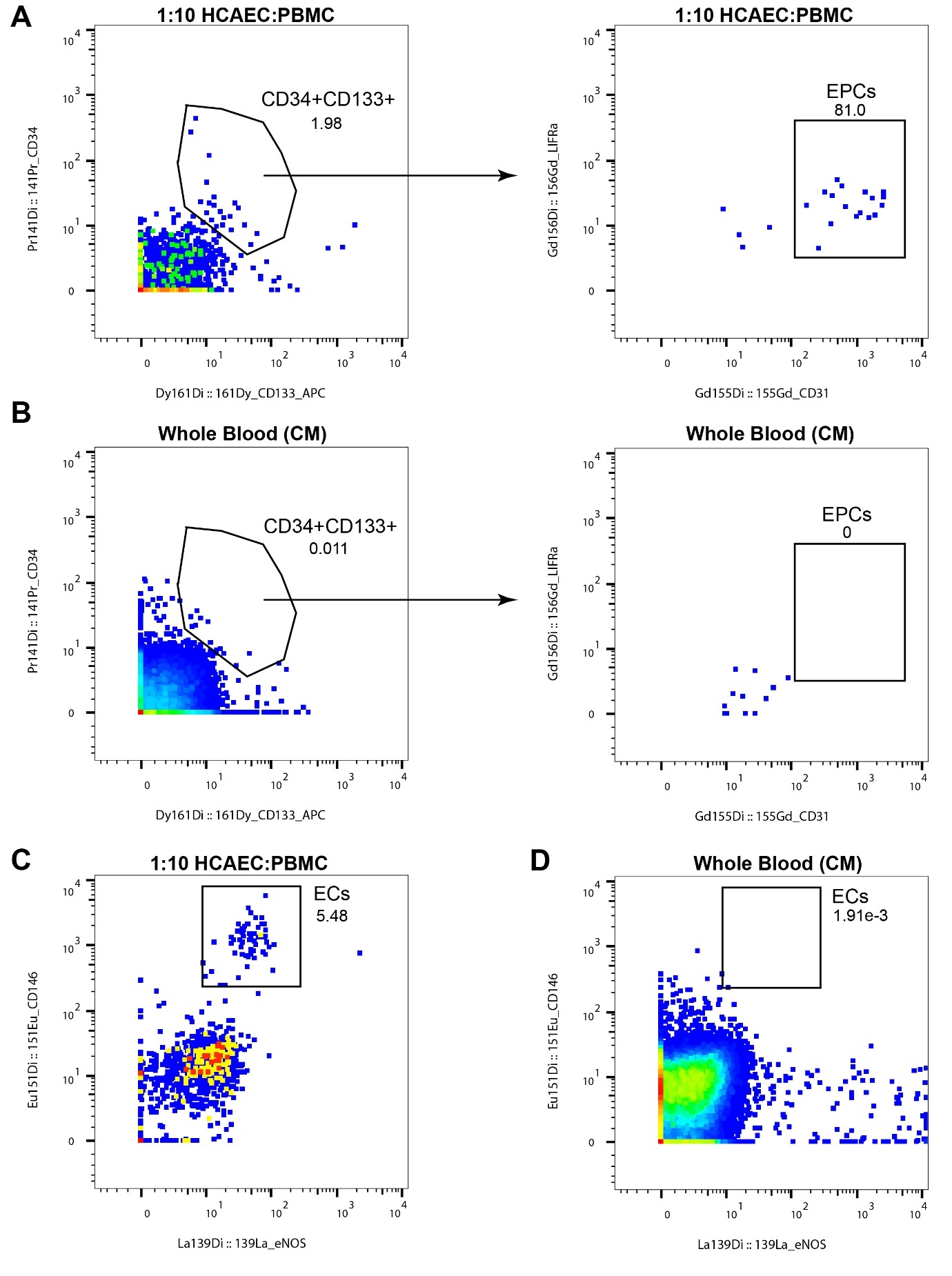


**Supplementary Figure 2.** Validation of antibodies marking circulating endothelial cells (CECs/ECs) and endothelial progenitor cells (EPCs) in fixed whole blood. Human coronary artery endothelial cells (HCAEC) were sourced from the laboratory of Prof Steven Wise at the Charles Perkins Centre, University of Sydney. These cells were spiked 1:10 into healthy PBMCs which were then stabilised with PROT1 (Smart Tube, Inc.) per manufacturer’s protocol. Whole blood from a chronic migraineur was similarly stabilised for comparison. **(A)** In the HCAEC/PBMC condition, EPCs were robustly detectable by gating for CD34^+^CD133^+^ and confirming with expression of LIFRα and CD31. **(B)** EPCs were not detected in whole blood without HCAEC cells. **(C)** CECs were detected by co-expression of endothelial nitric oxide synthase (eNOS) and CD146 in HCAEC cells, **(D)** but not in whole blood. We concluded that these endothelial cell types may not be robustly detectable in whole blood with the volume of sample viable for mass cytometry analysis, but proceeded with this strategy in experimental samples in case it was detectable in some migraineurs.


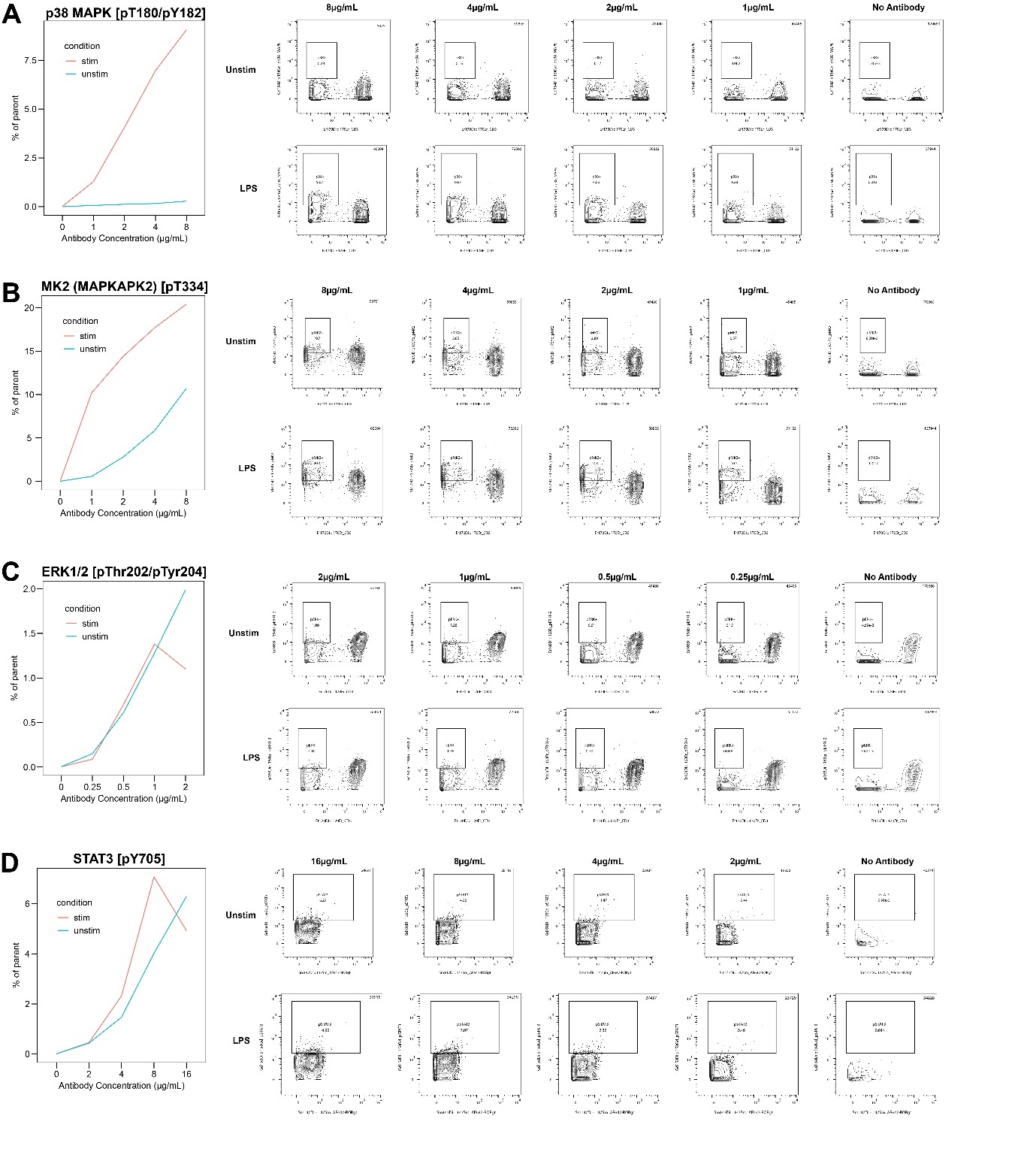


**Supplementary Figure 3.** Validation of select phosphorylated transcription factor-targeting antibodies. Human whole blood was stimulated with 2µL/mL lipopolysaccharide (LPS; eBioscience 00-4976-93) for 5 hours at 37˚C + 5% CO_2_. An unstimulated control was exposed to the same conditions in the same well plate, but LPS was not added. Whole blood was then collected from the well plate, fixed in PROT1 (Smart Tube, Inc.), and passed on for staining with gating surface antibodies and phosphorylated transcription factors. **(A)** p38 MAPK and **(B)** MK2 phosphorylation was drastically increased in the LPS stimulation condition. **(C)** ERK1/2 phosphorylation was, expectedly, not affected by LPS stimulation. **(D)** STAT3 phosphorylation was increased slightly by LPS stimulation, and was closely associated with RORγt expression, suggesting preferential expression in Th17 cells as expected. All antibodies demonstrated a concentration-dependent increase in staining intensity above background.


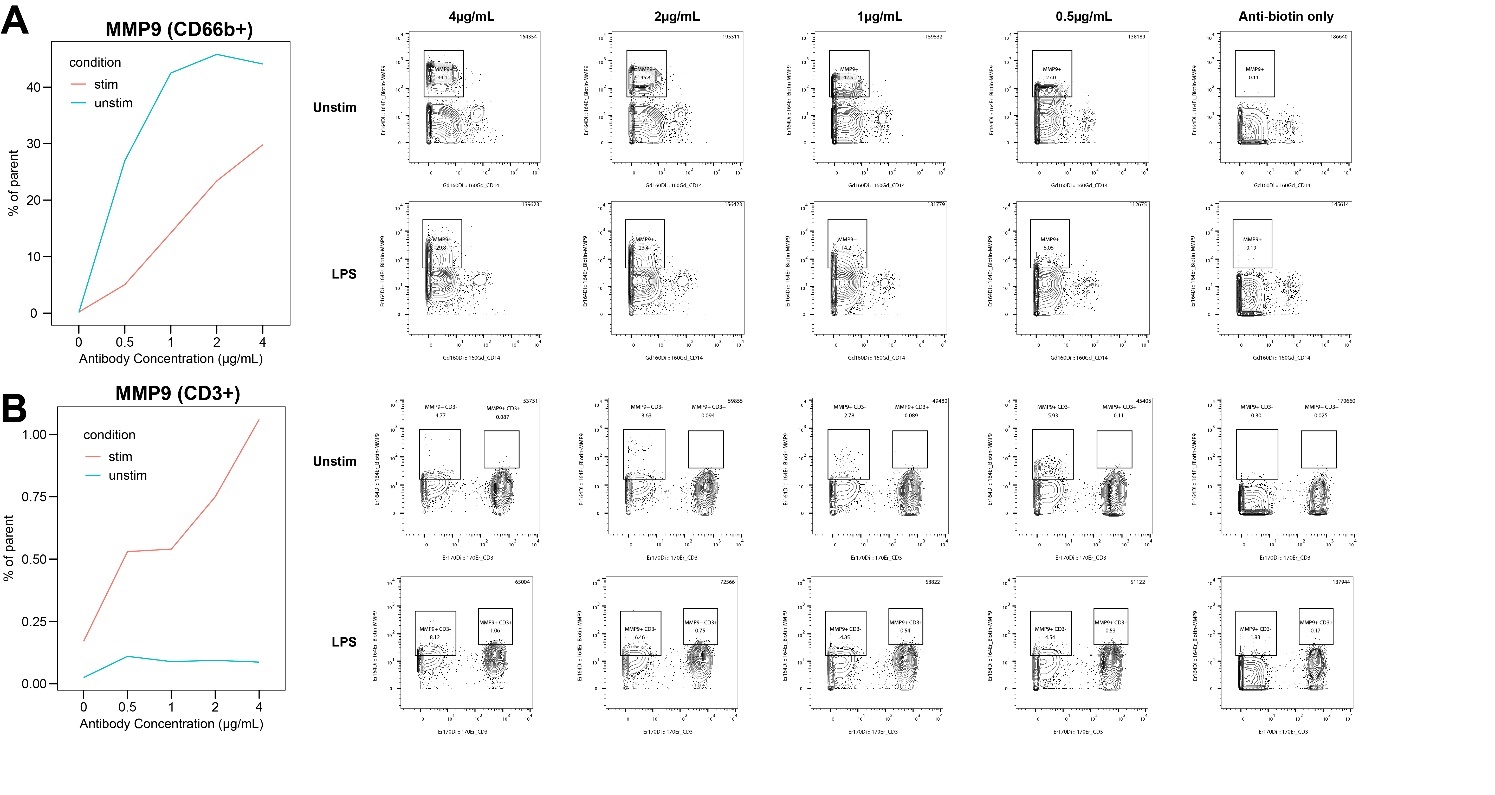


**Supplementary Figure 4.** Validation of MMP-9 antibody specificity. Whole blood was stimulated with lipopolysaccharide (LPS) as per the methods described in Suppl. Fig. 3. The unstained control received the metal-conjugated anti-biotin secondary antibody, but not the primary MMP-9-biotin antibody. **(A)** MMP-9 expression was substantially decreased in LPS-stimulated neutrophils compared to the unstimulated control, **(B)** but was markedly increased in LPS-stimulated CD3+ and CD3-CD19- cells. This behaviour is consistent with the known pro-inflammatory properties of MMP-9 in many cell types, and the specific tertiary degranulation mechanism known to be engaged in activated neutrophils and therefore possibly associated with extracellular MMP-9 release.

_
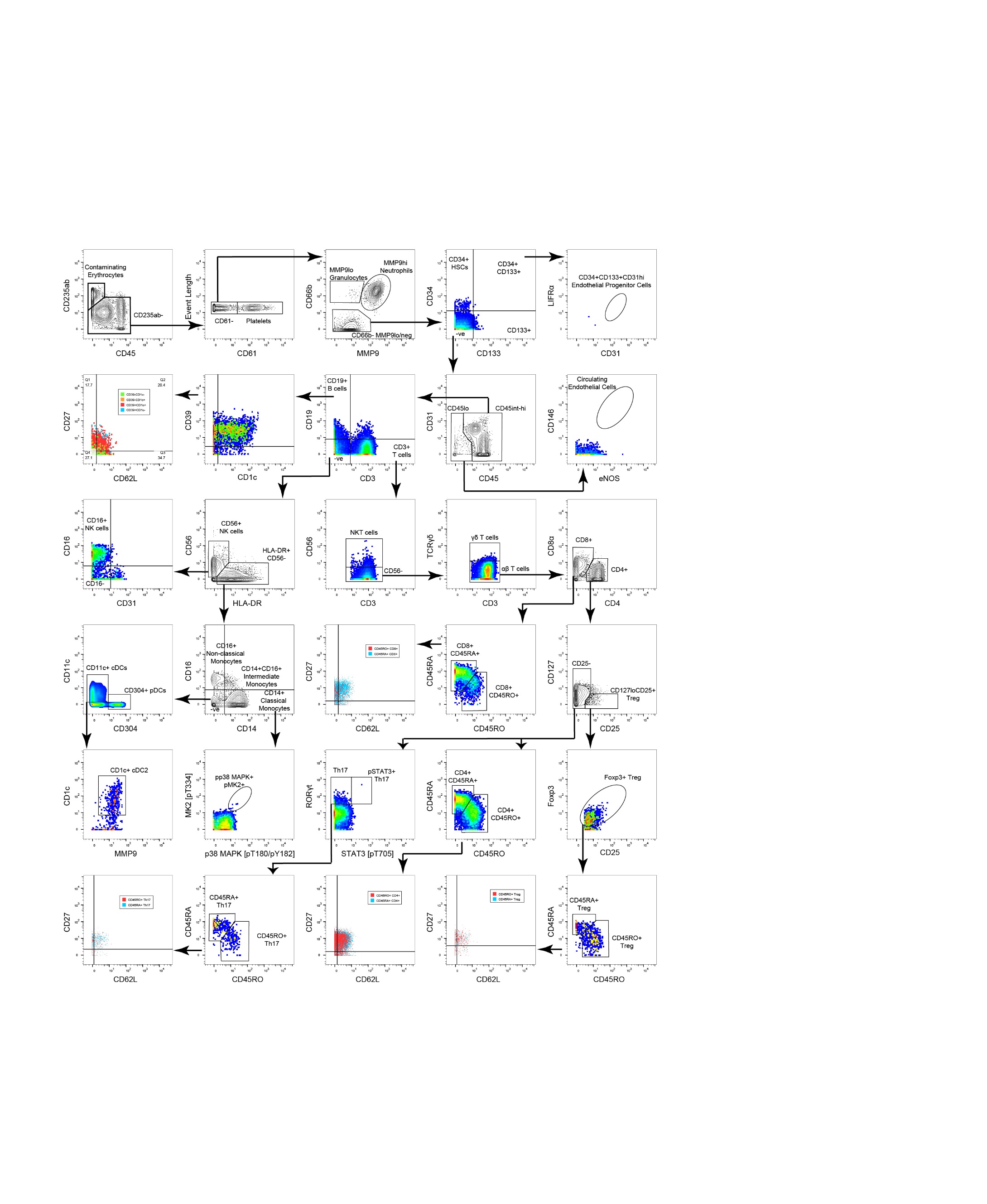
_

**Supplementary Figure 5.** Manual gating strategy for mass cytometry samples as applied to a representative participant. Each biaxial plot provides the expression values of two markers, subsets of which were iteratively passed down for further subsetting. Colours and contours indicate relative density of cells, except in those plots with an inset figure legend where the colours indicate ownership to the denoted parent gate. cDC: conventional dendritic cell; HSC: haematopoietic stem cell; NK: natural killer; pDC: plasmacytoid dendritic cell; Th17: T helper 17 cell; Treg: regulatory T cell.


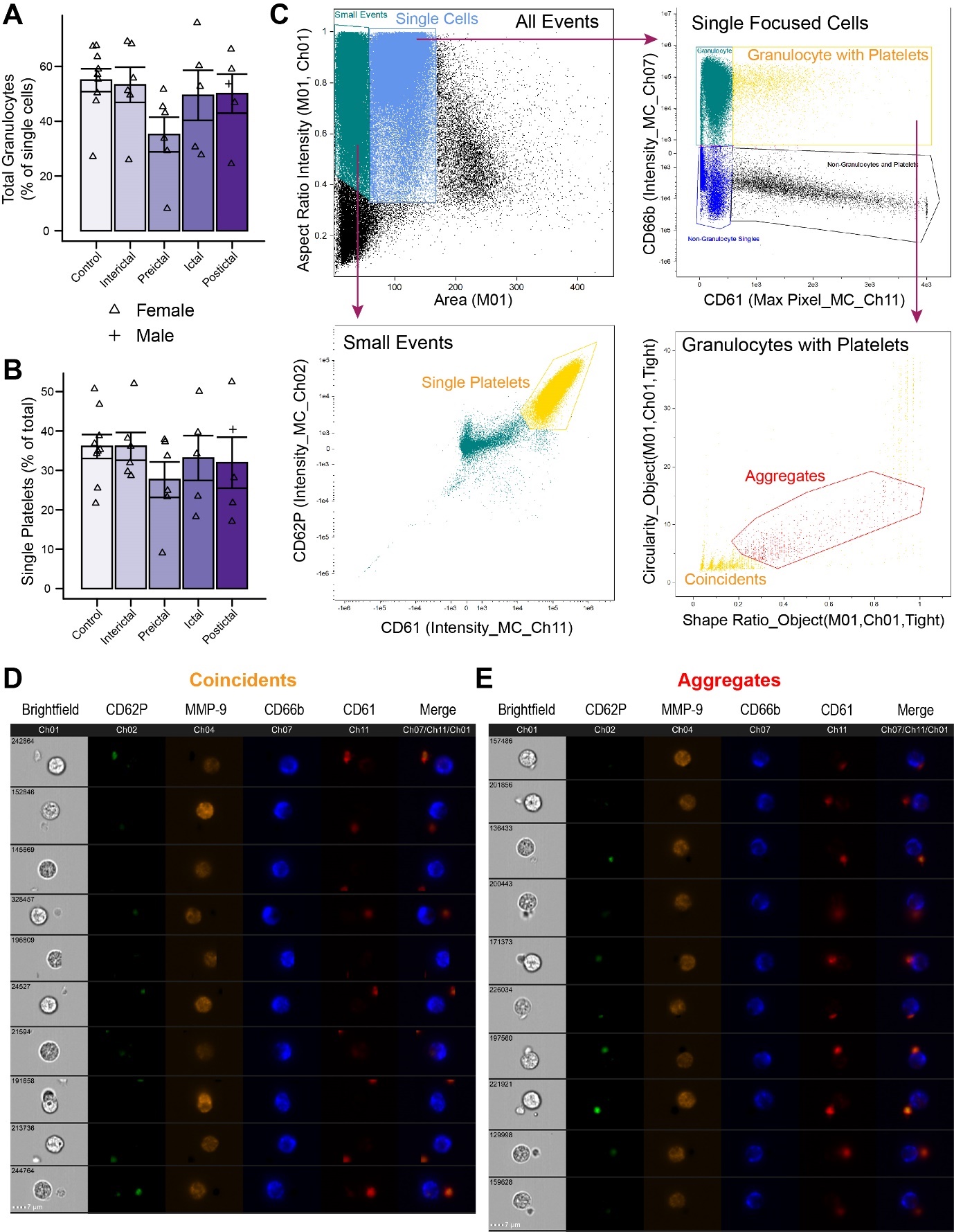


**Supplementary Figure 6.** Imaging flow cytometry of platelet-neutrophil aggregation. **(A)** The proportion of single granulocytes and **(B)** single platelets were not significantly different between, though there was a subtle decrease in the preictal period in line with the proportional increase in aggregates in this group. **(C)** The gating strategy used for isolating singlet and aggregate neutrophils and platelets. **(D)** The gating strategy achieved reliable isolation of coincident platelet-neutrophil events from **(E)** true platelet-neutrophil aggregates, in which the presence of CD61 (platelet marker), sometimes with coincidence of CD62P (P-selectin; platelet activation marker) was adjacent to a CD66b^+^MMP-9^hi^ neutrophil. Images are a representative subpopulation of the total.


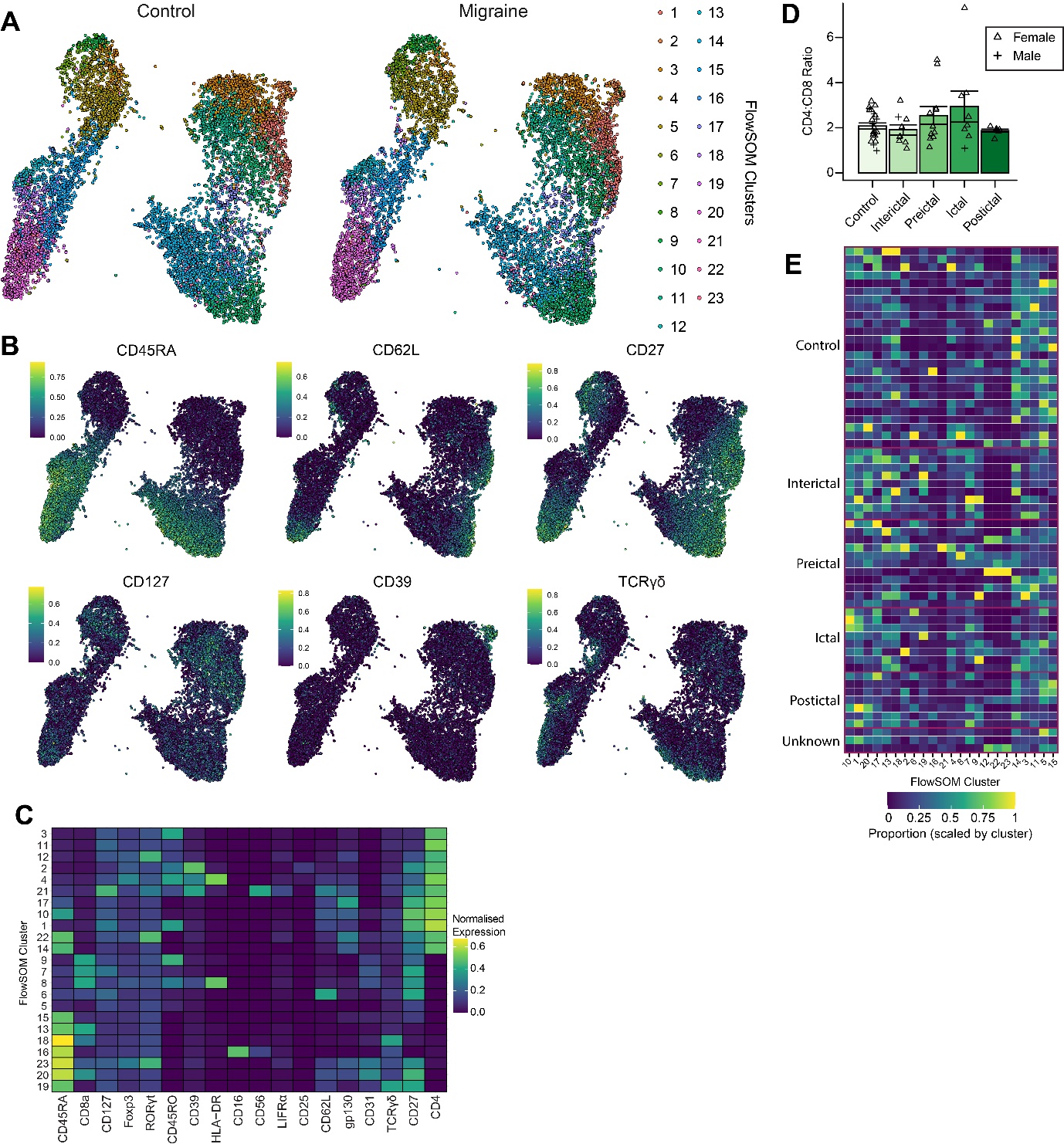


**Supplementary Figure 7.** Additional T cell results from mass cytometry data. **(A)** UMAP stratified by migraineurs and healthy controls shows some subtle variations in the T cell compartment between disease states. **(B)** UMAP of a selection of proteins whose expression was used to determine cluster annotations. **(C)** Heatmap of median expression of markers by FlowSOM metacluster. **(D)** The ratio of CD4^+^ to CD8^+^ T cells was not different between migraine phase conditions. **(E)** The relative abundance of FlowSOM clusters by individual participant. Participants are grouped by migraine phase. Proportions are scaled to the maximum percentage of total cells for each cluster. gp130: glycoprotein 130/CD130; LIFRα: leukemia inhibitory factor receptor subunit alpha.


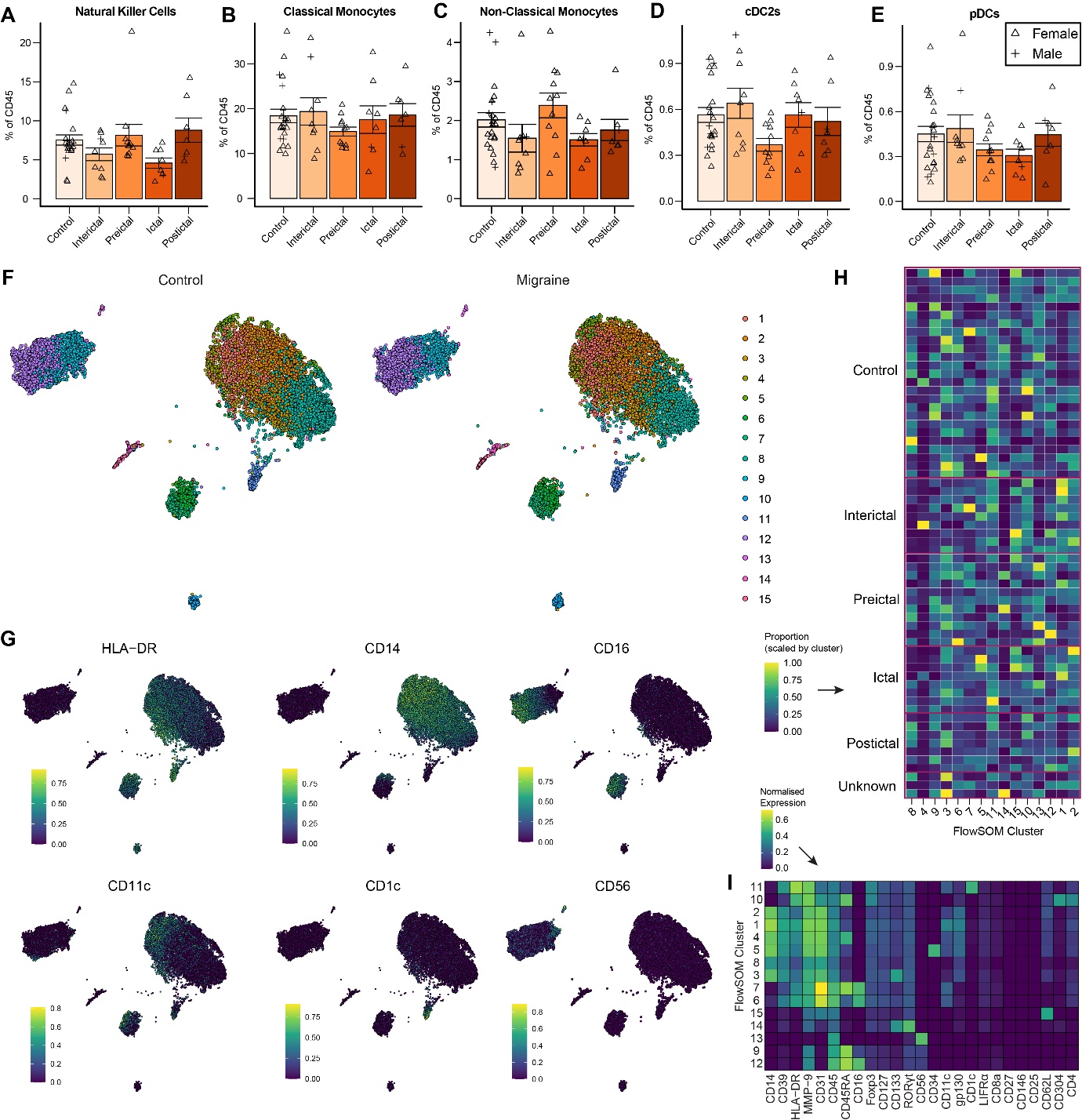


**Supplementary Figure 8.** Additional CD3^-^CD19^-^ myeloid and natural killer cell results from mass cytometry data. **(A)** Manually gated analysis of HLA-DR^-^CD56^+^ natural killer cell abundance showed no significant differences by migraine phase as a percentage of total CD45^+^ cells (*H_4_* = 8.89, *P* = 0.064). **(B)** There were likewise no significant differences in the abundances of total classical monocytes (*H_4_* = 1.90, *P* = 0.75), **(C)** non-classical monocytes (*H_4_* = 8.77, *P* = 0.067), **(D)** cDC2s (*H_4_* = 7.18, *P* = 0.13), or **(E)** pDCs (*H_4_* = 4.47, *P* = 0.35). **(F)** UMAP dimensionality reduction of downsampled (10,000 cells per condition) CD3^-^CD19^-^ cells coloured by FlowSOM cluster. Each dot represents a single cell. **(G)** UMAP representations of some key phenotypic markers for the major cell types in the CD3^-^CD19^-^ data set. Cells are coloured by expression of the indicated marker (scaled as a proportion of the maximum value). **(H)** FlowSOM cluster abundance for individual participants. Individuals are grouped by migraine phase. The heatmap is scaled according to the maximum frequency of each cluster. **(I)** Heatmap of marker expression by FlowSOM metacluster. cDC2: conventional type 2 dendritic cell; pDC: plasmacytoid dendritic cell.


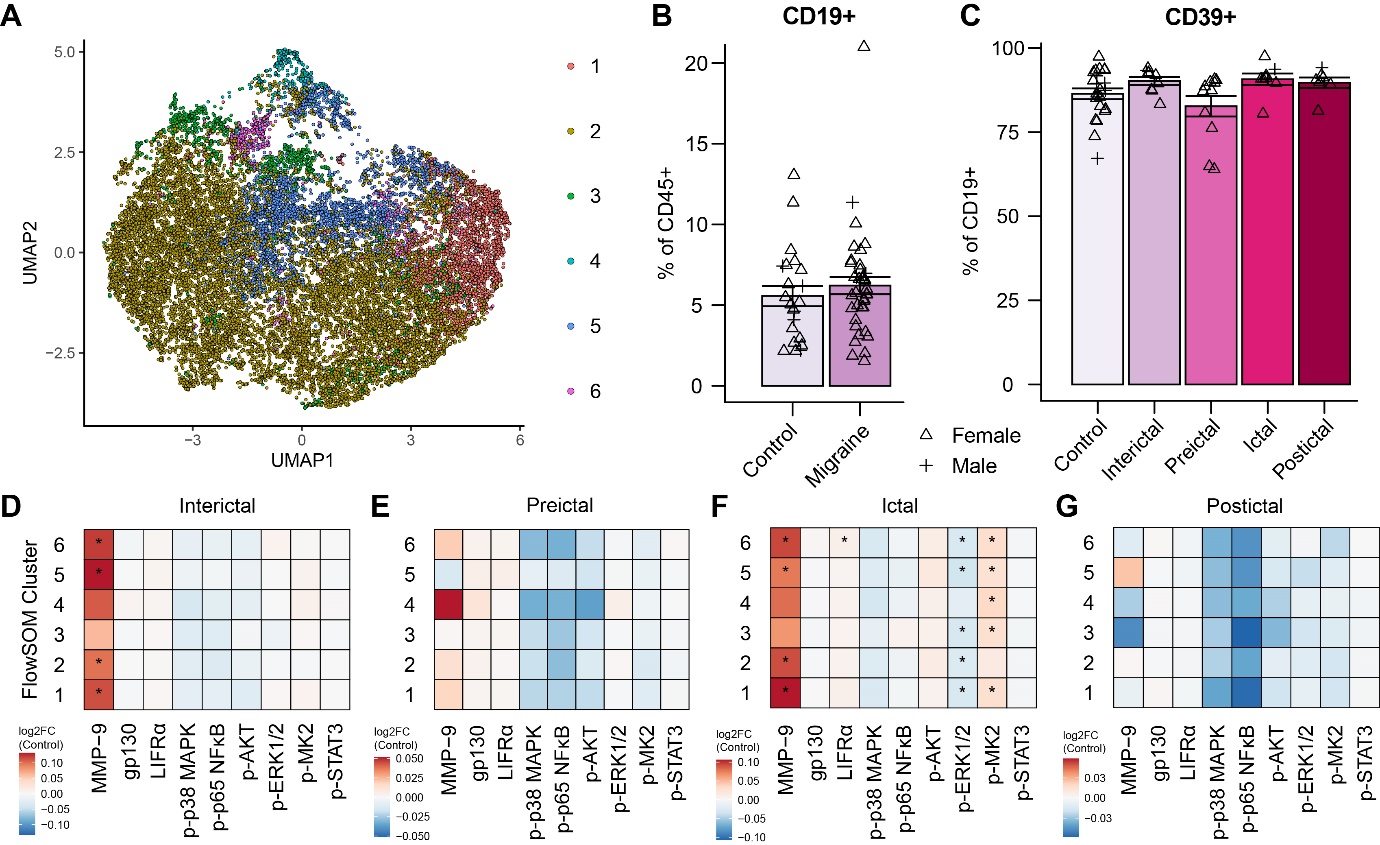


**Supplementary Figure 9.** Mass cytometry analysis of CD19^+^ B cells. **(A)** UMAP dimensionality reduction coloured by FlowSOM cluster. UMAP contains equal numbers of cells downsampled from both migraineurs and healthy controls. Each dot represents a single cell. **(B)** Manual gating analysis of CD19^+^ B cells as a proportion of all CD45+ cells found no significant difference between migraineurs and controls. **(C)** There were no migraine phase differences in the proportion of B cells expressing CD39. **(D)** Differential expression analysis of FlowSOM clusters found increased MMP-9 expression in multiple B cell clusters of interictal migraineurs compared to controls. **(E)** There were no expression differences for preictal migraineurs. **(F)** B cells of ictal migraineurs expressed higher levels of MMP-9 and phospho-MK2 in most clusters, and decreased phospho-ERK1/2 in most clusters compared to controls. LIFRα expression was increased specifically in cluster 6 (putative pre/pro B cells). **(G)** There were no expression differences in postictal migraineurs. AKT: protein kinase B; ERK: extracellular signal-related kinase; gp130: glycoprotein 130/CD130; LIFRα: leukemia inhibitory factor receptor subunit alpha; MAPK: mitogen-activated protein kinase; MK2: MAPK-activated protein kinase 2; MMP-9: matrix metalloproteinase-9; NFκB: nuclear factor kappa-B; p-: phosphorylated; STAT3: signal transducer and activation of transcription 3. **P* < 0.05.

**Supplementary Table 1.** Non-antibody reagents used for CyTOF sample preparation.

| **Reagent** | **Supplier** | **Cat #** |
| --- | --- | --- |
| Foxp3/transcription factor staining buffer set | eBioscience/Invitrogen | 00-5523-00 |
| Cell Acquisition Solution | Standard BioTools | 201240 |
| EQ Four-Element Beads | Standard BioTools | 201078 |
| Cell-ID DNA Intercalator | Standard BioTools | 201192 |

**Supplementary Table 2.** Reagent information for the CyTOF antibody panel. All metal-conjugated antibody reagents were custom-conjugated in house using MaxPar labelling kits (Standard BioTools). Fluorophore- and biotin-conjugated antibodies were purchased directly from the manufacturer. AF: Alexa Fluor; APC: allophycocyanin; BD: Becton Dickinson; CST: Cell Signalling Technology; IC: intracellular.

| **Label** | **Antigen** | **Clone** | **Supplier** | **Cat #** | **µg/mL** | **Stain Step** |
| --- | --- | --- | --- | --- | --- | --- |
| 89Y | CD11c | Bu15 | Biolegend | 337202 | 4 | Surface |
| 104Pd | CD45 | HI30 | Biolegend | 304002 | 8 | Barcode |
| 108Pd | CD45 | HI30 | Biolegend | 304002 | 8 | Barcode |
| 110Pd | CD45 | HI30 | Biolegend | 304002 | 8 | Barcode |
| 113In | CD56 | NCAM16.2 | BD | 559043 | 0.5 | Surface |
| 139La | eNOS | polyclonal | R&D Systems | AF950 | 4 | IC |
| 141Pr | CD34 | 581 | Biolegend | 343502 | 1 | Surface |
| 142Nd | CD19 | HIB19 | Biolegend | 302202 | 8 | Surface |
| 143Nd | CD45RA | HI100 | BD | 555486 | 1 | Surface |
| 144Nd | TCRγδ | B1 | Biolegend | 331202 | 8 | Surface |
| 145Nd | CD4 | RPA-T4 | BD | 555344 | 8 | Surface |
| 146Nd | CD8a | D8A8Y | CST | 81575SF | 8 | Surface |
| 147Sm | CD45RO | UCHL1 | Biolegend | 304202 | 4 | Surface |
| 148Nd | CD16 | B73.1 | Biolegend | 360702 | 2 | Surface |
| 149Sm | CD25 | M-A251 | Biolegend | 356102 | 1 | Surface |
| 150Nd | AKT (PKB) [pT308] | J1-223.371 | BD | 558316 | 1 | Nuclear |
| 151Eu | CD146 | PIH12 | BD | 550314 | 1 | Surface |
| 152Sm | CD66b | G10F5 | BD | 555723 | 2 | Surface |
| 153Eu | CD304 (Neuropilin-1) | 12C2 | Biolegend | 354502 | 2 | Surface |
| 154Sm | p38 MAPK [pT180/pY182] | 30/p38 MAPK | BD | 612281 | 8 | Nuclear |
| 155Gd | CD31 | WM59 | BD | 555444 | 0.5 | Surface |
| 156Gd | LIFRα (CD118) | 32953 | R&D Systems | MAB249 | 2 | Surface |
| 158Gd | STAT3 [pY705] | 4/P-STAT3 | BD | 612357 | 4 | Nuclear |
| 159Tb | CD235ab | HIR2 | Biolegend | 306602 | 0.5 | Surface |
| 160Gd | CD14 | M5E2 | BD | 557152 | 3.5 | Surface |
| 161Dy | APC | APC003 | Biolegend | 408002 | 4 | Surface 2^o^ |
| 162Dy | Foxp3 | PCH101 | eBioscience | 14-4776-8 | 6 | IC |
| 163Dy | CD1c | L161 | Biolegend | 331506 | 1 | Surface |
| 164Dy | Biotin | 1D4-C5 | Biolegend | 409002 | 4 | IC 2^o^ |
| 165Ho | CD61 | VI-PL2 | Biolegend | 336402 | 1 | Surface |
| 166Er | p65 NFκB [pS529] | K10-895.12.50 | BD | 558393 | 0.5 | Nuclear |
| 167Er | CD27 | M-T271 | BD | 555439 | 1 | Surface |
| 168Er | ERK1/2 [pThr202/pTyr204] | 4B11B69 | Biolegend | 675502 | 2 | Nuclear |
| 169Tm | Cy5 | Cy5-15 | Sigma | C1117 | 4 | Nuclear 2^o^ |
| 170Er | CD3 | UCHT1 | Biolegend | 300402 | 0.5 | Surface |
| 171Yb | CD62L | DREG-56 | Biolegend | 304802 | 4 | Surface |
| 172Yb | gp130 (CD130) | AM64 | BD | 555756 | 8 | Surface |
| 173Yb | MK2 (MAPKAPK2) [pT334] | P24-694 | BD | 562469 | 1 | Nuclear |
| 174Yb | HLA-DR | L243 | Biolegend | 307602 | 2 | Surface |
| 175Lu | CD39 | A1 | Biolegend | 328202 | 8 | Surface |
| 176Yb | CD127 | A019D5 | Biolegend | 351302 | 12 | Surface |
| 209Bi | CD45 | HI30 | Biolegend | 304002 | 0.5 | Surface |
| AF647 | RORγt | Q21-559 | BD | 563620 | 50µL/mL | Nuclear |
| APC | CD133 | Clone 7 | Biolegend | 372806 | 50µL/mL | Surface |
| Biotin | MMP-9 | D6O3H | CST | 15561 | 2 | IC |

**Supplementary Table 3.** Unsupervised analysis parameters for mass cytometry data. All events within the pregated were included with no downsampling for quantitative analysis; downsampling was only used for decrowding dimensionality reduction plots.

| **Pregate** | **Clusters** | **Metaclusters** | **Clusters used** | **Markers used for clustering and dimensionality reduction** |
| --- | --- | --- | --- | --- |
| **CD3+** | 10×10=100 | 30 | 23 | Foxp3, CD27, CD31, LIFRα, CD56, CD39, CD127, CD45RA, TCRγδ, CD4, CD8a, CD16, CD45RO, CD25, RORγt, CD62L, gp130, HLA-DR |
| **CD3-CD19-** | 8×8=64 | 20 | 15 | CD45, CD133, CD1c, Foxp3, MMP-9, CD27, CD146, CD304, CD31, LIFRα, CD14, CD56, CD39, CD127, CD45RA, CD4, CD8a, CD16, CD34, CD25, RORγt, CD11c, CD62L, gp130, HLA-DR |
| **CD19+** | 5×5=25 | 10 | 6 | CD133, CD1c, MMP-9, CD27, CD31, LIFRα, CD39, CD127, CD45RA, CD16, CD34, CD45RO, RORγt, CD11c, CD62L, gp130, HLA-DR |

**Supplementary Table 4.** Antibody panel for imaging flow cytometry experiments. AF: Alexa Fluor; BD: Becton Dickinson; CST: Cell Signalling Technology.

| **Measurement** | **Reporter** | **Clone** | **Supplier** | **Cat #** | **µL/test** | **Channel** | **Excitation Laser (nm)** |
| --- | --- | --- | --- | --- | --- | --- | --- |
| CD66b | BV405 | G10F5 | BD | 562940 | 5 | 7 | 405 |
| P-selectin | AF488 | AK4 | Biolegend | 304916 | 5 | 2 | 488 |
| MMP-9 | Biotin | D6O3H | CST | 15561 | 1.5 | 4 | 561 |
| …Streptavidin | AF568 | - | Invitrogen | S11226 | 0.5 |  |  |
| CD61 | AF647 | VI-PL2 | Biolegend | 336408 | 2 | 11 | 642 |
| Side scatter | - | - | - | - | - | 6 | 785 |
| Brightfield | - | - | - | - | - | 1,9 | - |

**Supplementary Table 5.** Cluster annotations for CD3+, CD3-CD19-, and CD19+ cells for clustering analysis. The ‘#’ column denotes the FlowSOM metacluster ID. NKT: natural killer T cell; Treg: regulatory T cell.

| **CD3+ (T cells)** | | **CD3-CD19- (Myeloid and NK cells)** | |
| --- | --- | --- | --- |
| **#** | **Annotation** | **#** | **Annotation** |
| 1 | CD4+ central memory | 1 | Classical monocyte (activated) |
| 2 | CD4+ CD39+ memory Treg | 2 | Classical monocyte |
| 3 | CD4+ effector memory | 3 | Classical monocyte (CD133^hi^) |
| 4 | CD4+ HLA-DR+ Treg | 4 | Classical monocyte (CD45RA^hi^) |
| 5 | CD8lo effector memory | 5 | CD14^+^CD34^+^ |
| 6 | CD8+ central memory | 6 | Intermediate monocyte |
| 7 | CD8+ central memory (transitional) | 7 | Non-classical monocyte |
| 8 | CD8+HLA-DR+CD39+ Treg | 8 | Classical monocyte (quiescent) |
| 9 | CD8+ effector memory | 9 | Natural killer (CD16^lo^) |
| 10 | CD4+ naïve CD27hi | 10 | Plasmacytoid dendritic cell (pDC) |
| 11 | CD4+ effector | 11 | Conventional dendritic cell type 2 (cDC2) |
| 12 | CD4+ memory Th17 | 12 | Natural killer (CD16^hi^) |
| 13 | CD8+ effector | 13 | Natural killer (CD56^hi^) |
| 14 | CD4+ naive | 14 | RORγt^+^CD133^+^ |
| 15 | CD8lo effector | 15 | CD62L^hi^HLA-DR^-^ |
| 16 | NKT CD56lo | **CD19+ (B cells)** | |
| 17 | CD4+ naïve gp130hi |  |  |
| 18 | CD8+TCRγδ+ | 1 | CD1c^+^ |
| 19 | Double-negative γδ T | 2 | B cell |
| 20 | CD8+ naive | 3 | CD133^hi^ |
| 21 | NKT CD56hiCD39+ | 4 | CD39^hi^CD27^+^ |
| 22 | CD4+ naïve Th17 | 5 | CD62L^+^ |
| 23 | CD8+ Tc17 | 6 | CD34^+^ pre/pro B cell |
